# Enhancing functional recovery for young people recovering from first episode psychosis via sport-based life skills training: outcomes of a feasibility and pilot study

**DOI:** 10.1080/21642850.2022.2147073

**Published:** 2022-11-21

**Authors:** Lauren E. Brooke, Daniel F. Gucciardi, Nikos Ntoumanis, Michael T. Chapman, Robin L. J. Lines, Yael Perry, Dylan Gilbey, Tegan Formby, Toby Phillips, Ashleigh Lin

**Affiliations:** a Curtin School of Allied Health, Curtin University, Perth, WA, Australia; b Curtin enAble Institute, Faculty of Health Sciences, Curtin University, Perth, WA, Australia; c Department of Sports Science and Clinical Biomechanics, University of Southern Denmark, Odense, Denmark; d Curtin School of Population Health, Curtin University, Perth, WA, Australia; e Telethon Kids Institute, The University of Western Australia, Perth, WA, Australia; f Ruah Community Services, Perth, WA, Australia

**Keywords:** Early intervention, functional recovery, life skills, first episode psychosis, sport

## Abstract

Early intervention within First Episode Psychosis (FEP) recovery efforts support functional recovery in several ways, including increasing levels of (1) physical activity (2) life skills, and (3) social connectivity. Sport has been proposed as an ideal platform to target these three goals simultaneously. The primary aims were to assess the feasibility of utilising sport-based life skills within FEP recovery efforts and test intervention components. The secondary aim was to evaluate the potential recovery benefits. Seven young people (aged 15–25 years) with FEP participated in a six-week sport programme alongside their support workers (community and peer workers) from the service, including peer workers with a lived experience of psychosis. The programme consisted of various sporting activities, which were designed to promote physical activity, maximise social connectivity, and teach life-skills (e.g. motivation, emotional regulation, and goal-setting) that are relevant and transferrable to other contexts (e.g. school, employment, independent living). The support participants engaged with the programme at the same level as the young people, with the role of providing support and normalising/modelling engagement. The young and support participants provided feedback during and after the programme via questionnaires and interviews. Young participants self-reported physical activity levels, psychological needs, recovery dimensions, and life skills pre- and post- intervention using established psychometric tools. We used thematic analysis to analyse the qualitative data and compared this information with other data collected (e.g. attendance, feedback, quantitative measurements). The study culminated with a process evaluation. The results indicated that, despite challenges with engagement for young people with FEP, sport-based life skills programming may be a feasible and useful recovery outlet. In addition, the results highlighted specific intervention components that were useful to promote engagement and recovery benefits. This study serves as a critical foundation for future sport-based work within FEP recovery.

A critical marker of recovery following a first episode of psychosis (FEP) is, in addition to symptom reduction, functional recovery. Early intervention efforts support functional recovery in several ways, including increasing levels of (1) physical activity, (2) life skills, and (3) social connectivity. Sport has been proposed as an ideal platform to target these three goals simultaneously (Brooke et al., 2019). Broadly defined, sport involves ‘physical exertion and skill as the primary focus of the activity, with elements of competition where rules and patterns of behaviour governing the activity exist formally through organisations and is generally recognised as a sport’ (May, 2021). In this way, sport is one of few contexts in which individuals can accrue physical activity alongside the development and/or refinement of cognitive, emotional, and behavioural skills that can be transferred to other life contexts (life skills; Gould & Carson, 2008) in socially rich settings that involve various social agents (e.g. coaches or instructors, other participants). Young people who have experienced FEP and their clinicians supported the potential of a sport-based, life skills programme for functional recovery (Brooke et al., 2020a). Informed by an intervention mapping framework (Bartholomew Eldredge et al., 2016), we developed the content and structure of such an intervention for implementation with young people with FEP (Brooke et al., 2020b). However, the idea that sport-based, life-skills can provide an innovative approach by which to maximise functional recovery for young people with FEP remains a thought experiment in the absence of evidence to optimise implementation and therefore potential efficacy and effectiveness.

The Medical Research Council (MRC) recommended that complex interventions be developed systematically, and any uncertainties be targeted through piloting (Craig et al., 2008). Hence, building on our preliminary work (Brooke et al., 2019, 2020a) the aim of the current study was to deliver and evaluate a feasibility and pilot study of a sport-based, life skills intervention for young people with FEP. In the current study, we present this intervention’s delivery, and report a process evaluation with the goal to inform future work.

## Methods

### Research context

This study was conducted in collaboration with an early psychosis functional recovery service (herein referred to as ‘the service’), from which we sought feedback throughout the design, implementation, and evaluation phases of the study. This service is located within the Perth metropolitan area but has a large catchment (over 900 square km.), including some semi-rural areas. During the final planning phases of the intervention (September 2018–February 2019), the service had just opened and begun accepting referrals from local early intervention services. Despite potential challenges associated with a new service with a large catchment, we selected this service as a partner for two primary reasons. First, the goals of the intervention and the service were well-aligned. Secondly, the service’s willingness and ability to dedicate time, support, and feedback throughout all study phases was critical to ensure that we achieved our study aims.

### Participants

The target population was young people (referred to as ‘young participants’ herein) aged 16–25 years who had experienced a FEP in the past 12 months, as diagnosed by a Consultant Psychiatrist, and were enrolled in the service. The service recommended enrolment of 5–15 young people for pragmatic reasons (e.g. capacity to transport participants from home to the session and having enough staff to provide mental health support during session if needed). Exclusion criteria included (i) inability to provide informed consent or complete the questionnaires/interviews due to insufficient language or cognitive capacity; (ii) being considered by the clinical care team as being unstable in symptomatology, unable to participate in physical activity, or to be a risk to self or others. Staff from the service (e.g. community workers and peer support workers, herein referred to as ‘staff participants’) were recruited to participate alongside the young participants to provide logistical and psychological safety support, and feedback on the program. Peer support workers are staff who have a lived experience of psychosis, and community workers are staff who work as the primary functional recovery care provider to clients of the service. There were no exclusion criteria for the staff participants. Seven young participants and nine staff participants took part in the study. All participants provided informed consent.

### Procedure

**Recruitment.** We conducted all recruitment through the service. Service staff referred eligible young people and arranged home visits (with the researcher and community worker) to obtain consent and complete the initial paperwork. During the home visits, young participants completed a physical activity screening questionnaire (ESSA, 2011) to assess readiness for physical activity. They also completed a wellness plan, which enabled the researcher to build rapport with prospective young participants, and to collect information about their specific needs, concerns, and goals. This information was used to maximise psychological and physical safety for the young participants, and foster engagement with the program.

**Intervention.** We ran a six-week intervention in which we used various sport activities (e.g. basketball, touch rugby) to promote physical activity, maximise social connectivity, and teach life-skills (e.g. motivation, emotional regulation, and goal-setting) that are relevant and transferrable to other contexts (e.g. school, employment, independent living). The programme was created through intervention mapping (IM), which involves a rigorous six-step process for intervention design (Eldridge et al., 2016). The full details of the IM process are reported in a separate publication (Brooke et al., 2020b). Three facilitators ran every session (two males and one female, ages 28–36 years). They all had experience in playing and coaching sport, sport programme delivery/development, and/or sport/exercise science or pedagogy, as well as master’s level qualifications in sport and exercise psychology. All facilitators completed a mandatory 1-hour training session in psychosis.

The programme was offered weekly for two hours in the afternoon at a local sport facility central to the majority of the service’s client base. The structure consisted of the following six phases: (1) welcome and ice-breaker activities (2) mental and physical warm-up (3) mental and physical skill learning (4) play/competition (with rules of the game catered to abilities), (5) mental and physical cool down, and (6) informal social time. A session plan outline can be found in the complete IM description (Brooke et al., 2020b), and complete session plans for the six sessions are provided in the supplementary materials.

Breaks were built into the session, and young participants were encouraged to take additional breaks when needed, such as watching from the sideline or engaging in alternative activities (e.g. journaling, ring toss). The sessions were designed to foster a gradual progression of comfortability and skills. For example, we supported physical skill progression by introducing skills in pairs or small group activities, and eventually moving to a large group game. Similarly, life skill progression included components like introducing goal-setting as it applies to step count for the session, and eventually progress to a discussion about goal-setting applicability to other aspects of life (i.e. ‘transfer’ being a defining feature of the definition of life skills; Gould & Carson, 2008). Lastly, efforts to support gradual social comfortability and skill development included, for example, starting in pair-based warm-up drills – pairs chosen by the participants – and eventually progress to whole group tag games with randomly-assigned teams. The sessions were designed to be flexible and iterative, and were adjusted at the moment (e.g. due to skills, numbers, weather, or engagement), and week-by-week in response to informal feedback collected from young and staff participants and the observations/reflections of the facilitators (‘good, better, how’ framework). The programme offered different sports (basketball, touch rugby, and field hockey), for two weeks at a time selected via a group vote every two weeks to promote autonomy (a critical component of life skills development; Hodge et al., 2012) and buy-in.

The primary focus of the sessions was on the sporting activities, with life skills development embedded in the activities by encouraging the young participants to apply the learned mental skills to other life contexts (e.g. breathing exercises learned in sport activity may be useful when feeling stressed in other contexts). This process was done through guided reflection in phase five of the sessions, and ongoing casual follow-up by the facilitators throughout the following sessions. Based on our needs assessment (Brooke et al., 2020a), we primarily targeted the life skills of motivation, confidence, and emotional regulation. Similar to related work on life skills (Hodge et al., 2012), motivation was targeted through the self-determination theory framework, with the goal of enhancing the participants’ sense of competence (e.g. goal setting, skill progression), relatedness (e.g. team building activities), and autonomy (e.g. choice of sporting activities; Ryan & Deci, 2017). Bandura’s (2001) social cognitive theory informed our efforts to foster self-efficacy via mastery experiences (e.g. guided reflection on overcoming challenges), vicarious experiences (e.g. observing others with shared experience), verbal persuasion (e.g. support from facilitators and other participants), and physiological/affective states (e.g. reframing, normalising, or controlling physiological states). Emotional regulation was targeted using social cognitive theory (e.g. reframing/controlling of physiological states through breath work), and through Gross (2015) extended process model of emotional regulation (e.g. modifying emotion-relation actions through breath work, or changing one or more aspects of the external world by choosing to take a break or engage in an alternative activity), and was supported by biofeedback (i.e. pedometers/heart rate monitors). Healthy snacks, water, and electrolyte sachets were readily available during sessions to promote safety and informal social interactions during breaks. In addition, communal food and drink were offered at the end during phase six to help further facilitate informal social interaction. Participants were encouraged to actively engage in the session, but not forced to participate in the activities if they were unwilling or unable to do so. They were also not required to engage in any data collection (e.g. questionnaires, interviews). Participants received an AUD 25 voucher for each session they attended as a reimbursement for time and travel expenses.

**Data collection**. Pre-intervention, young participants were offered the option to complete three additional pre-assessment measurements: (1) the International Physical Activity Questionnaire (IPAQ; Craig et al., 2003), selected for its utility in assessing physical activity levels in youth aged 15 and older, (2) the Basic Psychological Need Satisfaction and Frustration Scale (BPNSFS, Chen et al., 2015), selected to assess the satisfaction and frustration of the psychological needs for autonomy, competence, and relatedness, and (3) the Recovery Assessment Scale – Domains and Stages (RAS-DS; Hancock et al., 2019), selected for its ability to track mental health recovery outcomes. The measurements were given primarily to assess the feasibility of their inclusion in the intervention and subsequent impact on engagement, and as such were optional.

During the intervention, attendance and session engagement were recorded. Session engagement was recorded by facilitators for each young participant immediately post-session in a tabular format using facilitators’ recalled observations of young participants’ active participation (e.g. what session activities they did or didn’t engage in), interaction with others (e.g. chatting with facilitators, other participants, and/or support workers during the breaks), and outward appearance of enjoyment (e.g. celebrating sport victories, laughing). The facilitators recorded engagement notes post-session, as it was decided through feedback from the service that it would be more normalising for the facilitators to participate rather than take notes during the session. Feedback from young and staff participants was collected weekly (through phone calls, e-mail requests, meetings, and questionnaires) to assess primary outcomes. The first author collected all feedback and collated it in a tabular format.

Post-intervention, all participants were invited to participate in a semi-structured interview to gain insight relating to feasibility and specific intervention components. In addition, young participants had the option and complete post assessments (again primarily to assess their feasibility of inclusion in the intervention)The post assessments were the same as the pre assessments (IPAQ, BPNSF, RAS-DS) with the addition of the Life Skills Scale for Sport (LSSS; Cronin & Allen, 2017), selected for its utility in assessing the development of eight key life skills through sport (noting that we modified the instructional stem from ‘this sport has taught me to … ’ to ‘this program has taught me to … ’ to align with the nature of our study). Feasibility was further assessed by staff participants who provided feedback post-intervention via focus groups, separated into groups of community and peer support workers. Interview guides can be found in the supplementary material of the intervention mapping paper (Brooke et al., 2020b). The first author conducted all interviews.

### Outcomes

The primary outcomes were feasibility of the programme and assessment of intervention components. Feasibility was assessed through recruitment statistics, participant records (i.e. attendance, session engagement, and completion of questionnaire records for each participant), a record from the programme facilitators (i.e. exact session activities, reflections/observations, and modifications made), and feedback from young and staff participants. Intervention components were assessed through session engagement, session records, and feedback from young and staff participants. Secondary outcomes included life skills development, physical activity levels, social engagement levels, and psychosis recovery progress. Meaningful change in secondary outcomes was not expected due to duration and sample size. These outcomes were measured via the optional measurements pre- and post-intervention, and via semi-structured interviews post-intervention.

### Analysis

We utilised Moore et al. (2015) Medical Research Council (MRC) guidance for process evaluation in addition to Bowen et al. (2009) framework for feasibility studies as an overarching guide for the analysis of the results and to inform a process evaluation as a core component of our feasibility assessment. We used the following components from the MRC guidelines to direct our evaluation: description of intervention and its causal assumptions, implementation, mechanisms of impact, and outcomes and context (see Moore et al., 2015). Using Bowen et al. (2009) framework, we evaluated the following intervention facets: acceptability, demand, implementation, practicality, adaptation, integration, expansion, and limited efficacy. Within both frameworks, for each component we assessed the following: the relevant questions, study findings, and questions for future work. This framework instructed the entire data analysis and as such will be woven through the results, but the culmination of it will be presented in the discussion. Interview data were transcribed verbatim and analysed in NVivo (version 11; QSR, 2010) using Braun et al.’s (2016) thematic analysis, which involves a six-step iterative process: (1) familiarisation with data, (2) initial code generation, (3) generating initial themes, (4) theme review, (5) theme definition and naming, and (6) report production. This method was chosen because the reflexive and flexible nature of it allowed us to capture the unique experience of the various participants, which was important for the feasibility assessment and any potential impact on secondary outcomes. Participant interviews were analysed separately by group; domains and themes were combined where appropriate. The theme generation process was more deductive in nature as it sought to answer predetermined questions set by the process evaluation framework, however the reflexivity of thematic analysis enabled the researchers to also include inductive elements. In addition, the reflexivity and flexibility of this approach were especially important in constructing themes that captured the experience and feedback of different participant sets (i.e. young person, community worker, and peer support worker), and then connecting the interview data to the other study data. As such, after step five and before step six (report production), we compared the results of the thematic analysis with the other study data, namely, feedback collected weekly from participants, the observations of the facilitators, questionnaire results, and the intervention records (i.e. recruitment, attendance, engagement, modifications, and measurement participation records), to identify any relevant patterns or contrasting evidence.

## Ethics statement

The research presented and reported in this paper was conducted in accordance with the National Health and Medical Research Council National Statement on Ethical Conduct in Human Research (2007) – updated in March 2014. The research study received human research ethics approval from the North Metropolitan Health Service – Mental Health – Human Research Ethics Committee (EC00273), approval number 13_2016; and the Curtin University Human Research Ethics Committee (EC00262), approval number HRE2018-0748. All human participants gave written informed consent.

## Results

Thematic analysis of the interviews resulted in the generation of the following domains: enablers, barriers, recovery benefits, skill development and transfer, and recommendations. Relevant themes and sub-themes constructed for each domain are summarised in Table 1. With the view to integrate interview results with other data sources, we present the findings as they relate to the following intervention phases (and related components): (1) research context and recruitment; (2) attendance and engagement (including session attendance and engagement, measurement/feedback attendance and engagement); (3) barriers and enablers to recruitment, attendance and engagement (including personal, environmental, and logistical; (4) recovery benefits and skill development/transfer; and (5) programme modifications and recommendations. Each of the five sections will contain subheadings that refer to the domains and sub-domains generated from the interview results (see Table 1), and the corresponding themes will be illustrated with quotes and other relevant data from multiple sources.

**Table 1.** Domains and themes constructed from thematic analysis of participant interviews.

Domain	Sub-domains	Themes
Enablers	Personal	• Internalised motivation through alignment with goals (i.e. getting out of the house, social engagement, motivation, PA).
	Environmental	• Safe Environment (i.e. supportive, non-judgemental, and normalised) made possible through: o Structure (progression, breaks, normalising components) o Facilitators (welcoming, engaging, normalising) o Staff participants (rapport, peer support awareness, modelling, normalise/ balance power differential, safety)
	Logistical	• Logistical components made attendance possible and alluring (i.e. transport support, provision of food/drinks, reimbursement)
Barriers	Personal	• Place in recovery journey not conducive to attendance o Relative approach is important in interpreting engagement
	Environmental	• Unappealing environmental conditions (i.e. large group, sport type)
	Logistical	• Logistical factors made engagement challenging (i.e. timing and length of program)
Recovery benefits	N/A	• Increased social interaction • Connection with others who share a common experience • Opportunity to challenge counter-productive beliefs • General mental health (i.e. enhanced mood, distraction from challenges) • Increased anxiety management • Increased confidence • Increased motivation for physical activity • Non-attendance benefits (i.e. self-assertion, motivation for other recovery outlets)
Skill Development and transfer	N/A	• Enhanced emotional regulation • Enhanced/application understanding of motivation • Use of skills outside of program
Recommendations		• Longer program • Youth-friendly elements • Population sensitive equipment • Established service partner important

### Research context and recruitment

At the time of recruitment, the service partner had 17 active clients; 11 met the study’s eligibility requirements, seven of whom signed up and provided consent to take part in the study (see Table 2 for demographic information). Of the six who were ineligible, two were too unwell to participate, and four were still undergoing assessment. Four eligible clients did not sign up because of clash with other commitments, or because they disliked exercising. In addition, nine staff from the service agreed to participate in a support role alongside the young participants, and to provide feedback during and after the intervention. They held varying roles at the service: community workers with primary clients (*n* = 4), administration manager (*n* = 1), peer support worker (*n* = 3), and social work student (*n* = 1). Feedback from service staff regarding aspects of the recruitment process is presented in Table 3, where they highlighted some of their and their clients’ biggest concerns about (e.g. transportation) and draws to (e.g. social opportunities) participating in the study.

**Table 2.** Demographic information of young participant.

Part #	Age	Accommodation	Marital status	Highest Education	Current work/.education	Current sport	Past sport
1	23	live with partner	gf/bf >3mo	year 11	none	no	yes – basketball
2	18	family home	single	year 12	none	no	yes – martial arts and swimming
3	18	family home	single	TAFE	none	no	yes – basketball, swimming, tennis
4	18	family home	single	year 11	none	no	yes – swimming, Ironman, Australian football, Rugby
5	19	family home	single	year 11	none	yes – basketball 2 h per week	yes – basketball
6	18	family home	single	year 12	University – part time	no	no
7	22	family home	single	not reported	part time; 20 h a week	yes - basketball; 8–10 h per week	yes-basketball

Note: Gender is not presented for confidentiality reasons. 5 males and 2 females participated.

**Table 3.** Qualitative responses from community workers (*n* = 5) regarding referring clients to the study.

Question	Support worker response (frequency)
When you talked to your clients about the study, what interested them the most?	• Reimbursement (3) • Social opportunities (2) • Opportunity to partake in a larger group setting (1) • The range of different sports (1) • Chance to participate in an activity to break the cycle of boredom (1)
When you talked to your clients about the study, what were their biggest concerns?	• Transport/location/getting there (3) • Socialising in a larger group (1) • Looking silly in front of other young people (1) • Confidence (1) • Being the biggest person there (weight) (1) • Timing of programme (1)
What made you most interested in referring your clients to the study?	• Opportunities for socialisation/social skills development (5) • Opportunity for physical activity/exercise (3) • Opportunity to build confidence (1) • Mental health benefits of sport (1) • Importance of opportunities to get out of the house (1)
What were your biggest concerns in referring your clients to the study?	• Location/transport issues (1) • Motivation issues (1) • Anxiety (1) • That they would only attend once (1) • Regular Cannabis use (1)

### Attendance and engagement

**Session attendance and engagement.** Of the seven young participants, five enrolled in the study before the sessions began, one enrolled after the first session, and one enrolled after the second session. The average attendance rate for the young participants was 46.9%. A display of young person attendance and reasons for not attending can be found in Table 4. There were at least two community workers at every session, and at least two peer support workers at all sessions except one. Overall, our facilitators observed engagement levels across all sessions were high for each activity, with the group warm-up and group game activities drawing the highest levels of perceived engagement. Some young participants and staff chose to sit out or engage in alternative activities at various points (although this was not the norm). Overall, the facilitator’s reflection notes show increased levels of social interaction as the sessions and intervention progressed over the six weeks.

**Table 4.** Attendance records of young people and reasons for absences.

Part #	Session 1	Session 2	Session 3	Session 4	Session 5	Session 6
1	Yes	Yes	Yes	No – reported feeling unwell and needed to sleep	Yes	No – reported feeling unwell and needed to sleep
2	Yes	Yes	No – driving lesson took priority	Yes	Yes	Yes
3	Yes	Yes	Yes	Yes	Yes	No – had medical procedure day before and was unable to attend
4	No – reported not feeling up to it	Yes – (big accomplishment to attend as reported by support worker)	No – anxiety and avoidance (as reported by support worker)	No – anxiety and avoidance (as reported by support worker)	No – waiting for a call (case manager suspects anxiety and avoidance)	No – no longer engaging with service
5	Yes	No – sore knee	Yes	No – family visit took priority	No – reported gym injury from previous day	No – family time took priority
6	n/a	No – university work took priority	Yes	No – university work took priority	No – university work took priority	No – unwell (hospital admission)
7	n/a	n/a	Yes	No – other sport training took priority	No – will no longer be part of the programme as it clashes with work and training obligations	No – n/a

**Measurement and feedback engagement.** Four of the seven young participants completed the questionnaires pre-intervention, of whom three completed the measurements post-intervention. These same three were the only young participants to engage in a post-intervention interview (others did not respond to invitations to be interviewed). These three young participants also had the highest attendance rates (see Table 4). Young participant feedback engagement during the intervention was low. Two young participants twice responded to feedback requests via phone, and one responded to a feedback request via e-mail once. All community and peer support worker participants participated in a post-intervention interview (one focus group for each). Staff participants provided feedback via phone interviews and group meetings after the first session, via team meetings after the second session, and via questionnaire for sessions three–six. Collecting feedback through meetings led to feedback from more staff participants, as only 2–3 staff participants completed the feedback questionnaires each week. However, the staff participants provided more in-depth feedback via the questionnaires than via meetings or phone interviews.

### Reported enablers and barriers to recruitment, attendance, and engagement

In the interviews, young and staff participants reported personal, environmental, and logistical enablers and barriers to attendance and engagement. Feedback and observations collected throughout the intervention corroborated these findings.

**Enablers**

***Personal****.* A discussion between the young person and service staff about the program’s alignment with young person’s recovery goals fostered recruitment, attendance, and/or engagement.

If it was in line with their goals and what they wanted to achieve, and that was getting out and being more active, socialising with people their own age, then it was easy to say ‘Oh, great we’ve got this great program, do you want to give it a go and see if you can meet some of them?’ (community worker)

The young participants reported that this alignment with recovery goals (for example, increasing physical activity, having a sense of belonging, or meeting others with similar experiences) is what motivated them to attend the program, and that this motivation was strengthened by pursuing these goals in a fun way. One young person put it this way:

Personally, I think it was getting me out of the house to do something physical, because I’ve struggled in that, with motivation in general. I was thinking I could be losing weight doing this, so it’s a motivation for me. But it’s also fun so you don’t think about weight. I’m looking at my body saying am I losing weight excessively, so I think for me it was very important. I loved the sport component, just having fun. I loved seeing the people, I loved just talking with [the facilitators] and sport people.

Another young participant said:

I was motivated for a sense of belonging, participation as well. I enjoyed the sports in general, like rugby, and basketball, and getting involved, getting to know people, getting to make new friends, and learning more about people.

***Environmental.*** The participants reported that the environment of the programme contributed greatly to their attendance and especially the engagement levels of the young participants. All participants reported that a strength of the programme was that the environment was inviting. More specifically, participants reported that it felt safe, supportive, non-judgemental, and normalised; this perception was created through the structure, the facilitators, and the presence of staff.

*Structure.* The structure of the sessions was important to foster engagement levels and ensure future attendance. Participants reported that the structure supported the young peoples’ varying abilities, interests, recovery goals, and fitness levels, which put them at ease. As one peer worker described:

I was a little bit anxious going into it, but I think you guys made it quite comfortable and I didn’t … the anxiety sort of went away quite quickly because I realized that you guys are kind of walking us through it. We weren’t just expected to be awesome at it. Yeah, it was comfortable, and it was guided.

Young participants also reported that the structure put them at ease as it was predictable:

It was all planned and structured out, so we knew exactly what was going to happen in the program and how it was going to go about.

The progression of varying activities seemed to allow the participants to warm up slowly (physically and psychologically) to the sport of the day, and to the other participants. As one community worker described:

Yeah, understand the game, the rules, and just a different activity throughout so it wasn’t the same thing the whole time. Having regular breaks as well, having that broken up. I also really liked the skills element. They’re looking at motivation and goal setting before and after. It has an intention, how it kind of supports you, or what can you get out of today that would be of most value to you. Getting them to make short term goals and focus on the positives of doing something like this.

The breaks, alternative activities, and food and drink provided enabled the participants to listen to their needs, and to engage in a way that served them. As one community worker said:

They were able to just go to the side and just take a minute and fill up their drink bottle and join back in in their own pace which I reckon was really good. Breaking it up made it more accessible for people who maybe weren’t so good at the particular sport.

The young participants expressed the importance of the structure in making them feel safe.

It was important to do the introduction and the warm-up and everything like that. Just by letting everyone know who each other was, you feel safer knowing and stuff like that.

The participants expressed that certain elements of the structure helped to normalise the environment. For example, all participants expressed that they thought it was beneficial that the facilitators and staff participants participated alongside the young participants, and that no distinctions were made, or special treatment given:

Everyone was sort of treated the same, and it was all sort of equal. It created that sort of safe space environment where they’re like wow, I can just be myself, I can enjoy this sport and it’s not about me having experienced psychosis or having mental illness, this is just about me enjoying the sport and getting out and doing the things, and building that confidence (peer support worker).

In addition, the presentation of different session components helped to normalise the environment. Participants reported that the sessions seemed to meet the needs of the young participants, without feeling too mental health specific. For example, as one peer worker described:

I remember one thing is when we were doing the breathing activity, you didn’t make it psychosis specific, and you made sure to mention that, ‘oh, we do this because athletes use it as well’. That sort of normalised the experience.

One young participant also expressed how normalised the sessions felt:

It didn’t become about kids in recovery for me. I thought it was just a bunch of adults at sport. To be honest it’s just a chilled recreational community sport thing. I didn’t even think of people’s mental health issues. It was more about we’re all there to have fun.

Similar to the feedback from the participants regarding the programme structure, facilitator reflections also noted that the progression of the activities (i.e. graded social interaction and activity levels, and social ice breaker and non-sport-specific games) seemed to help foster engagement levels.

*Facilitators.* The participants reported that the facilitators played a critical role in creating an environment that was inviting (i.e. safe, supportive, non-judgemental, and normalised). Participants reported that the facilitators were approachable, easy-going, and nonjudgmental, which in turn put the participants at ease and fostered engagements. The community workers reflected that the facilitators made the young participants feel comfortable and supported:

Even if a young person was sitting on the side, one of the facilitators would sit out, and actually have a conversation with that young person. One I’m thinking about in particular, that young person really valued that experience more so than anything else from the program that this male took time to speak with him in a positive way.

The young participants’ reflections supported this notion:

The facilitators were great. To be honest I was nervous they would be judgemental about people in general who are maybe overweight and stuff. When I saw them, I was like, ‘sh*t they’re sporty’. But when we met, they literally put me at ease, and were joking with me pretty much every session. Yeah, and it got real chilled because I think you just talk about TV shows and general stuff and then you all become friends.

Lastly, participants noted that the facilitators played an important role in normalising the environment:

I noticed that the facilitators talked to some of the staff and were just like … ‘oh, how’re you going, what happened on the weekend?’ But then you also do the same for the young people. Like, you just turn around and do the same for the young people. I think that would have been a very … don’t know the word for it … just normalising the entire experience in that it’s not like all the conversation is towards the staff and we’re talking about you behind your back, because I know that … for some people who experience psychosis, paranoia is very prominent and that there may be that fear of even like lingering paranoia that’d be like, are people talking behind my back? What’s happening here? But I think because everyone was so open and welcoming of everyone that came in, it was quite lovely. (peer support worker)

*Staff Participants.* Young and staff participants reported that the inclusion of the staff participants helped foster an inviting (i.e. safe, supportive, non-judgemental, and normalised) environment. The staff participants were originally included in the design to provide logistical support (e.g. transport), promote psychological safety, and to provide feedback. However, the reflections of the young and staff participants and the facilitators showed that the inclusion of staff participants had unexpected benefits. The staff participants reflected that participating alongside their clients strengthened their rapport and relationship with the young participants:

Yeah, I think just normalising that this is a learning experience for most of us, in terms of some of the sports, definitely helped in building a rapport I think. But also allowed us to reflect back to the young person, ‘Oh, you did this really well, you seemed happy when you did this activity’, or, ‘Tell me what that was like for you, because for me it was like this. I want to check in if that was all right for you.’ It actually allowed a conversation around experience and potentially implementing some of the things we’ve been trying to work on anyway. That shared experience, that that would have been present in that conversation. I think it’s more value than I was just watching them on the sidelines and being like, ‘You didn’t even participate, how would you know?’ If they were challenged by something especially you could have a conversation around that and it was heard because you did the same thing (community worker).

Interestingly, this rapport extended beyond the immediate worker–client relationships, and into the service as a whole:

I think it helped build our therapeutic relationships, not just with the people we’re working directly with, but with the wider young participants, and as our service goes more into making groups and stuff I think it builds better relationships across the service for all of us. Young people that we saw individually, young participants that maybe we’d only heard about in team meetings, we could all interact together, and support each other, and so it brought all of us together. I thought that was really nice (community worker).

The staff participants also noted how participating alongside the young participants helped level the power differential between them. Most of the young participants had more sporting experience than the staff participants, and as such the sessions provided opportunities for the young participants to take on a helping or leadership role. The staff participants expressed the value in the opportunity for the young participants to help others:

That sort of thing, I think it just shows them that they have more value than they think they do, and that they have important things in their life that they can offer to other people. (community worker)

The young participants also focused on the feeling of safety that the inclusion of staff brought to the intervention:

It made me more at ease. Because if you put a whole bunch of (young people with psychosis) together, you don’t know what the heck’s gonna happen and (the staff) can deal with it. Even though nothing really happened it was good to have that there.

The facilitators’ reflection notes stressed the importance of the staff participants in modelling and normalising engagement, especially in the early sessions. The facilitators observed that having a consistent base of people willing to try an activity regardless of their skill or comfort seemed critical in maximising the buy-in of the young participants. They also noted that it was helpful to have a consistent group of people to balance numbers on days when young participant numbers were low. Lastly, the facilitators were pleased to observe the growth in the rapport between the young participants and staff as the sessions progressed.

***Logistical.*** The participants also reported that the logistical provisions (i.e. transport, refreshments, and participant reimbursement) of the intervention allowed for attendance and engagement. First, transport was critical for attendance. All young participants were transported to all sessions by the staff participants, and staff reached out to the young participants weekly to confirm their attendance and to plan pick-up. All participants confirmed that without this support, the young participants would have most likely not attended. Proximity of the young participant’s home to the intervention location did not alter this response. In addition, the participants reported that the refreshments provided during and after the session encouraged attendance and engagement. Not only did the food promote social interaction and help the young participants feel supported, but it also made it physiologically possible for the young participants to participate, as they felt that the food and hydration helped support them through the demands of physical activity.

Lastly, the participants reported that the reimbursement provided to young participants at each session served as an incentive to sign up and to attend. However, although the vouchers seemed to support recruitment (see Table 2), the motivation to receive the vouchers was discussed much less than other motivational factors (e.g. opportunity to work toward recovery goals), especially as the sessions went on.

**Barriers*.*** Barriers are those factors reported by the participants and/or observed by the facilitators that inhibited young participants from signing up, attending, and/or engaging in the intervention sessions.

***Personal.*** The reported personal barriers to all phases of the intervention related largely to the young peoples’ recovery journey. Some young people were too unwell to be ready to engage at the recruitment phase. Lingering symptoms and ongoing poor mental health prevented young people from attending (see Table 4). We reported in the enablers section that the intervention’s alignment with recovery goals supported engagement throughout the program. Conversely, the service staff reported that, for some young people in their care, if intervention didn’t align with their recovery goals or was not well suited to their current recovery level, engagement suffered as a result.

I think it’s about sort of where they’re at in their recovery journey. There’s a window, where it’s like really useful for where they’re at, but some people might be at a point where they’re at really low motivation, and they just don’t want anything to do with it.

You might get people on the opposite side of the spectrum who are actually doing really well and have been in recovery for a while. They’ve met some of their goals, and they’re actually working on some of their own personal goals in their own time, and that’s like the reason why they’re not coming to the group, because they’re actually working on things that they want to do with their social networks and physical health networks, stuff like that. It’s trying to find the best fit. (peer worker)

The staff participants also emphasised the importance of taking a relative approach when looking at attendance and engagement overall.

I think for some as well that it’s their experiences from the past and how they see the world definitely impacted engagement. Maybe there’s no control in their life and their level of commitment generally is quite low. The level of commitment maybe is not their own drive, but other people’s. So they’ve tried it once and they’re satisfied with that. That is a huge achievement going once for some young participants. (community worker)

***Environmental.*** Although some people reported that the group environment and mix in sport options were strengths in the intervention, staff participants reported that these elements also served as barriers for some young participants. The large group environment was reportedly overwhelming to some and may have limited attendance and engagement. In addition, if the sport of the day was not the young person’s sport of choice, they were less likely to attend.

***Logistical.*** Certain logistical components served as barriers across all phases of the intervention. The timing of the intervention served as a barrier in the recruitment phase, as at this time the client numbers were low and the service was still assessing some of their new clients. Transport was a common concern of young participants during the recruitment phase, but this was alleviated through the service providing transport. However, the community workers reported that the timing of the intervention made it difficult to support the young participants in arranging their own transport.

I think a longer program would be beneficial. Especially in a way that we can have more opportunity to promote independence within the young participants, in terms of doing off their own back and taking some real onus of the situation in the groups, I think a longer group would have given you more of an ability to start working on those. For example, doing transport training with them and getting them onto public transport, doing that with them, supported. And then them doing it themselves. Because I know a lot of them wouldn’t have attended if we didn’t drive them or pick them up.

In addition, some of the young participants were at a point in their recovery where they had returned to university, work, or other commitments, which served as a logistical barrier to recruitment and attendance (see Table 2).

### Recovery benefits and skill development and transfer

The study was a pilot and feasibility study, and as such the primary outcomes were assessing the feasibility and acceptability of using sport for a population with FEP, and to test out intervention components. Perceptions of potential efficacy (or benefits) to participants is an important consideration for feasibility studies; if participants perceive little or no value then they are unlikely to remain engaged in a program.

**Recovery benefits.** The participants reported some recovery benefits for the young people because of their engagement in the study. First, the participants discussed the benefits of social interaction in general.

It helps in general social situations. It’s funny to say I went to a sports situation and I gained social skills. But you do because you interact with many different people. My job actually I do, but my job I’m in a formal setting. This is different, this is social, every day for me so I think it was really important. (young person)

The social component was very powerful, because that could be the start of their journey back to socializing, essentially. For me, it was a couple of different things, but like this might be the beginning of someone’s journey back to socializing, because that might help build their confidence, that little bit, and to help them move forward even further. (peer worker)

More specifically, the participants also reported positive benefits from the opportunity to connect with others who share a common experience.

Yeah, I think it just made them reflect on maybe how isolated and kind of caught up they had been with their own experiences, but actually there’s other young participants going through this and it’s great to normalize that experience but also talk with people your own age. (community worker)

The young participants reported that connecting to others going through a similar experience gave them ‘a sense of belonging’ and an additional support network. The participants also reported that being a part of the study gave the young people an opportunity to challenge debilitating or counter-productive beliefs.

I think it was a chance to challenge some of their anxious thinking and what they expect will happen. Having actually an experience where you can challenge them on and say, ‘Well, actually this didn’t happen here’, or how you were thinking about that beforehand didn’t actually eventuate. If you think of some of your other kind of thoughts are potentially wrong. Just allow a conversation after that experience to happen. (community worker)

Some of the participants reflected that participating in the study helped support their general mental health and manage or distract from some of their symptoms or challenges. One young participant described a shift in their mood:

So I think I was going through other triggers and mood swings so I was worried this was gonna show ‘cause they can be extreme, but it wasn’t and I found that being in a controlled environment actually helped me. I made a difference and that was what I really liked about it, I was happy for one. During that time, you know when you get up and you feel happy about things? I actually started feeling happy about something, going to something.

The participants expressed that participation helped lessen anxiety in that in creating a positive experience in a situation in which young people with FEP generally felt anxious. One young participant expressed how this anxiety management extended to other areas of their life:

Having this (program) then helped me do other things like go out and go to the shops by myself and do stuff by myself a lot more. Even though I was doing that all before, it made me not scared to do it, if that makes sense. Not as anxious.

Similarly, the staff participants emphasised the impact the study had on the young participants’ confidence.

A lot of people who have experienced psychosis, afterwards they’ve dropped out of education and all their friends are off doing these other things and that leaves them being quite isolated, and the longer they stay isolated the less confidence and lower their self-esteem becomes. I think being able to have those leadership roles and be in a group where things are a level playing field helps build that up again to being back into maybe the biggest group they’ve been in for the past year for some of them. Realise actually it’s okay, it’s not a big scary thing, everyone was friendly, I participated in a group, 12, 15 people, had a good time, and maybe none of their concerns or anxieties around what that would actually be like, came through. I think that reinforces them in other parts of their life to be like, ‘Actually why am I putting these things off? I can go out there and join in this group or start to follow this hobby instead of just being at home’ (community worker).

In addition, the participants all expressed that the study helped support the young participants’ motivation for physical activity:

I think certainly for the young participants it really helped with their motivation. Our clients are quite young, clients are 17 to 24ish, so maybe they don’t have the insight that you get as you get a bit older about physical activity. I think sport is just a really good way to bring it and make it fun.

Lastly, the community workers reported that there may have even been benefits for those who were enrolled but chose not to attend, in that it gave the young participants an opportunity to assert themselves, or engage in other activities that best served their current needs.

I think even the ones that started to say that they weren’t coming in the weeks I think it gave them an opportunity to put in their own boundaries around what they want in their life. ‘Actually I don’t want to turn up this week and I’ve made that choice, I’m not going to do it just because I might offend. (my support worker)’

Another community worker said:

I think as a positive it made (a young person) realise that ‘actually I do want to get out, actually I do want to do a bit more activity’. One of the main reasons they stopped attending is because they joined a gym, start attending that three times a week and saw that as a priority over the study. That had been something we had tried to work on before they had done the study, and I feel that maybe the study was a kick start for them.

*Measurements.* Young participants were invited to complete self-reports of psychosocial factors pre- and post-intervention. These measurements were offered with the aim to assess willingness to complete, as significant changes could not be expected in the time frame or with the sample size. However, for the three young people who completed all measurements (who were also the three with the highest attendance rates), the measurements show a favourable trend. We focus on the qualitative data here; interested readers can request to view the survey results.

**Skill development.** Components of the study included physical and life skills training. In regard to physical skills development (e.g. sport skills, fitness), the participants did not report a change in their physical skills beyond the previously discussed shift in perceptions of sport/physical activity, and the accompanying shifts in confidence, motivation, and anxiety. However, the participants did express a shift in some of the targeted life skills, such as breathing and goal setting:

Learning about motivation and the different types of motivation, the good types, the bad types. I found the breathing as well, the breathing helps. It’s really calming, the techniques that you’ve thought of.

**Skill transfer.** A critical component of life skills training is the successful transfer of the skill to another context. One young person said:

I just think the strategies I learnt there without even knowing and then reflecting on my change, I’ve managed to put to other situations and how to calm myself and the breathing when I’m anxious. Like for example, with driving, I think I need to put aside that anxiousness and the fear of failure because I need my license. But if I had trouble I was asking Mum in a more calmer tone and not freaking out ‘cause I now know not to freak out when you’re anxious.

Speaking about a different young person, one community worker reflected:

(Young person) said that what they got from it was it helped support their motivation to do other things outside of the study, so they started volunteering twice a week, which wasn’t happening before so that’s a really good outcome for them. They’ve also found benefits in looking at their motivation, like the extrinsic and intrinsic motivation. They found that, I think quite helpful.

### Programme modifications and recommendations

General feedback for future programs was overwhelmingly positive. Nevertheless, the participants provided some constructive feedback that may be informative for future work. First, it was suggested by all that a longer programme would enable more opportunities for skill development, recovery benefits, physical health benefits, and more independence (e.g. transport) for the young participants. Second, participants emphasised the importance of more youth-friendly elements in the programme methodology, such as tablets with emoji-based Likert scales for some of the measurements, and colourful and customisable workbooks (vs. the plain black workbooks used in the study; see session plans). Third, the participants recommended enhanced sensitivity to the population’s need in regard to equipment, specifically in regard to larger bib sizes and wrist-based heart rate monitors (vs. under the shirt chest monitors). Finally, the involvement and support from the service was critical to the successful running of the study from logistical (e.g. transport), implementation (e.g. service staff participating alongside young participants), and evaluative (e.g. providing feedback during and after) perspectives. A service with an established client base may have enhanced recruitment and retention, in turn enabling a more favourable support worker-to-young person ratio. However, a more established service may also have more competing demands (e.g. a higher client load may limit the service’s ability to provide such hands-on support). Full details of the feedback are provided in Table S1 of the supplementary material.

## Discussion

In this study, we documented the delivery and evaluation of a sport-based life skills programme designed to meet the needs of young people recovering from FEP. This study builds on our previous work in which we proposed that sport may be an innovative context in which to target the complex and urgent needs of young people recovering from FEP (Brooke et al., 2019), assessed the barriers and enablers to implementing this notion into practice (Brooke et al., 2020a), and developed an intervention framework utilising the rigorous process of intervention mapping (Bartholomew Eldredge et al., 2016; Brooke et al., 2020b). The next logical step of this building process was to take the evidence-based idea from paper into the field. Accordingly, the aims of the study were to assess the feasibility of this type of programme for the population, test intervention components, evaluate if future work in this area is warranted, and provide recommendation for such work if so.

As a feasibility and pilot study, the results are vast. Moore et al. (2015) Medical Research Council (MRC) guidance for process evaluation and Bowen et al. (2009) framework for feasibility studies provided an overarching framework for analysing, interpreting, and discussing the results. A detailed display of the process evaluation is provided in Tables S2 and S3 in the supplementary material. Overall, the evaluation indicated a high degree of acceptability for sport-based recovery work within FEP, suggest that this work may be a feasible and beneficial recovery outlet for the population, and provide valuable insight into critical intervention components. Overall, young and staff participants’ feedback indicated that the programme was engaging, enjoyable, and beneficial. The feedback, coupled with recruitment and attendance records, indicated that the programme may be feasible on a larger scale, but that further piloting is warranted to address specific challenges (e.g. recruitment and retention) and uncertainties (e.g. working with a more established service, adapting to other locations/cultures). Feedback from young and staff participants, in addition to reflections from the facilitators, gave valuable insight into specific useful intervention components, suggesting that the structure (e.g. graded participation), amenities (e.g. snacks, breaks), and personnel (e.g. friendly and engaging facilitators, staff participating at the same level) were critical to the success of the program. Although the results do not provide enough evidence for a full trial, these results urge further piloting in this area. A comprehensive list of suggested future research questions developed in the process evaluation is provided in Tables S2 and S3 (e.g. what regions, services, and/or phases of recovery are most in demand of such a program? How can the support from the service be maximised, but the burden be minimised?).

This study addresses a crucial gap in the literature and is novel several ways. It is well documented that early intervention following a FEP is crucial for reducing functional impairment (e.g. social and occupational) later in life and overall improving the trajectory of one’s quality of life (e.g. Albert & Weibell, 2019; Fusar-Poli et al., 2017). While the management of psychotic symptoms and co-morbid psychopathology is a critical part of early intervention protocol, efforts to enhance functional recovery are also important (Santesteban-Echarri et al., 2017). Paramount functional recovery goals in FEP include increasing physical activity (e.g. Shannon et al., 2020), building life skills (e.g. Allott et al., 2020), and promoting social connectedness (e.g. Ludwig et al., 2021). Most published interventions to date target one or two of these goals (e.g. Lambden et al., 2018; Watkins et al., 2020). However, further functional recovery efforts that maximise (already limited) engagement are needed. This study targets these three goals simultaneously, addressing this gap in a novel way. First, this study is novel in that it is, to our knowledge, the first to utilise intervention mapping (Bartholomew Eldredge et al., 2016) within FEP research. The participants’ feedback indicated that the study was successful in meeting and adjusting to the specific needs of the population, for which we credit the use of this method. Second, this is the first intervention study that we are aware of to apply sport-based life skills development within FEP functional recovery efforts. To date, researchers have focused predominately on the benefits of exercise (e.g. strength training, see Fouhy et al., 2020) for FEP physical health recovery. However, the additional benefits of sport (e.g. life skills development), demonstrated to be useful in other vulnerable populations (see Hermens et al., 2017; Sampogna et al., 2022), have yet to be explored for people with FEP. In addition, this study is novel in that it specifically targets physical activity, social connectivity, and life skills development in one integrated intervention. The integration of multiple recovery outcomes in one study may be a valuable option for FEP functional recovery efforts, considering the challenge of engaging young people with FEP (e.g. Brown et al., 2019; Woodhead & Monson, 2013). In other words, targeting multiple recovery outcomes within one intervention (e.g. the current study; Curtis et al., 2016) may maximise the limited time that young people do engage. Lastly, this study is innovative in its use of mental health support staff (e.g. community workers and peer support workers) as participants alongside the young people with FEP. Previously researchers have found running an intervention directly through a FEP service to be useful for recruitment and retention (e.g. Curtis et al., 2016), but to our knowledge, this is the first to engage service staff as study participants who engage on the same level as the young people with FEP and assist them to engage in the program. Of particular interest is the inclusion of peer support workers with a lived experience of psychosis, who provided a unique and valuable perspective. We hope that this feasibility and pilot study is a starting point for these novel intervention components, and that future research explores these concepts further.

This study is not without limitations. The low participant numbers, short intervention duration, inability to collect more objective outcome data, and inability to implement randomisation to experimental groups yielded inconclusive information regarding the secondary outcomes (i.e. functional recovery benefits). In addition, the feedback from the staff participants indicated working with a new service may have negatively impacted recruitment and retention (e.g. no established relationships between young people and service); piloting with a more established service would be useful. Next, we report results and make inferences regarding feasibility only from a small sample of participants who engaged with our data collection processes; thus, we’re unable to know whether these perceptions are consistent with or different to those participants who engaged with the programme but not the interview process. Finally, for future work, a larger sample size and the inclusion of information such as ethnicity, duration of untreated psychosis (DUP), socio-demographic information, and clinical presentation would give confidence to representation of true FEP cases, and allow for generalisability. We do not believe that these limitations should deter future work in this area, as this study was merely a first of its kind starting point for sport-based life skills functional recovery efforts in FEP.

In conclusion, the results of this study suggest that sport-based life skills programming may be feasible and useful within FEP functional recovery efforts. We found that, despite the challenges of engaging this population, further work in this area is warranted; we implore researchers and clinicians to consider building upon the current study.

## Supplementary Material

Supplemental Material
