# Supplementary material for: Enhancing functional recovery for young people recovering from first episode psychosis via sport-based life skills training: outcomes of a feasibility and pilot study
Source: Health Psychol Behav Med. 2022 Nov 21;10(1):1136–58. doi: 10.1080/21642850.2022.2147073 (PMC9683043; doi:10.1080/21642850.2022.2147073)
Supplement: Supplemental Material [file RHPB_A_2147073_SM7104.zip › HPBM-2022-0007.R2_supp_session plans.docx]

| **Session 1: Basketball (indoor)** | | | | |
| --- | --- | --- | --- | --- |
| *Key Session Outcomes:* 1) Building rapport; 2) Physical activity; 3) Social interaction; 4) Skill development / confidence  *Materials needed*: basketballs (10-20), ball pump, cones (30), bibs (20), first aid kit, alternative activities (e.g., ring toss, skittles, beach bats), water, snacks, speaker  *Note:* Facilitators are to treat all participants and support workers equally; support workers and facilitators participate alongside young people for all activities | | | | |
| PHASE | TIMING | KEY COMPONENTS TO TARGET | ACTIVITIES | KEY TEACHING/ FACILITATING POINTS |
| INTRODUCTION ICE BREAKER(S) | 20 min | - set expectations - build comfort/familiarity with group and program - get to know names | - **Acknowledgement of country:**    - “We wish to acknowledge the traditional custodians of the land we are meeting on, the Whadjuk people. We would like to acknowledge the strength, resilience and capacity of Noongar people in this land.” - **Introduction** (introduce facilitators) - **Housekeeping**   - toilets, water, snacks, other activities, breaks, structure of session - **Ice breakers**    - 1. this or that: create a line on the court with cones, present opposing preferences and have people go to one side of the line or the other depending on their preference (dogs or cats; vanilla or chocolate; coffee or tea; Eagles or Dockers; summer or winter; sunrise or sunset; hamburger or hot dog, tomato or tomato; rover or ocean; Nutella or peanut butter)   - 2. ball pass/name game (say name and fav sport/team) - **Ground rules/expectations** (group discussion with whiteboard; have group come up with list and prompt if any of the below aren’t discussed)   - Respect   - Listen to your body/needs   - Support others   - Give it a go/challenge by choice   - Come in the right mind (sober) - **Set Up**   - watches   - workbooks (name on outside; inside: one thing you hope to get out of the session; # of steps you think you might do in today’s session) | Run by 1 facilitator, others participate  Facilitators to treat all participants and support workers equally; support workers and facilitators participate alongside young people for all activities |
| PHYSICAL WARM UP | 10 min | - elevated heart rate - movement in major muscle groups - social interaction/familiarity - priming for physical skills required for day (e.g., hand-eye coordination, reflexes, spatial awareness) | - **Dynamic movement and activities**    - Pairs:     - HSNT/Cone: (best ⅔): In pairs with 1 cone per pair. Facilitator calls out “head, shoulders, knees, toes, cone” in random order. Participants are to touch different parts on their own body when called. When “cone” is called, participants compete to grab the cone first.     - Tennis ball reflex challenge: In pairs with 2 tennis balls per pair. Partners face each other 1.5 arm length apart. One partner holds 2 tennis balls out in front at shoulder height with arm extended; other partner stands with hands behind back. Partner with balls randomly drops 1 ball; goal is for other partner to catch ball before it hits the ground. Challenge; drop 2 balls at once   - Group:     - Chasing game: “Bib Tag”. Two players attempt to catch group members (‘runners’) by throwing a soft bib they each possess. If a player is caught they stand ‘stuck’ at that point until another runner releases them by tapping them on the shoulder.     - The area played in is relative to the size of the group allowing enough space for players to move safely without collision. (e.g., 10 players = 25m x 25m area).     - Progression of the game - Once a runner is caught, instead of becoming stuck they go to pick up a spare bib and join the initial tagger(s) in catching remaining runners.   *To adjust the challenge the following adaptations were made where needed:   - - - For those struggling:       - Inclusion of rest zone to the side of playing area that could be used when needed         - Provide individual players with a small cone that may be used as a shield to deflect bibs that are thrown towards them.     - For those striving:       - Constraining their movement style. (e.g., instead of running freely, they must sidestep or race walk)       - Providing a ‘balance hat’ in the form of a small cone. The player must keep this on their head without using their hands whilst moving around the area. | Run by 1 facilitator, others participate or engage with those on sideline |
| (BREAK) | 5 min | To allow participants to catch their breath, get a drink or a snack, and engage in informal social interaction. Facilitators are to engage participants in casual conversation (water, electrolytes, snacks available). | | |
| SKILL LEARNING | 20 min | - Shooting - Passing - Dribbling | **Basketball confidence meter: “**How’s your basketball confidence?” Create a line of cones going from red to yellow to green; have participants and facilitators line up according to their basketball confidence/experience- red being low confidence/experience; green being high.  **Passing introduction**: Players are each numbered from 1 to *n* within small groups (e.g., 4-6 players). Players start by passing in order (i.e., 1 to 2, 2 to 3, etc.) Players are then instructed to move ball around in a chest pass, bounce pass, and overarm passing techniques.  Progression: Instructions are then called out that all groups follow while still passing. (1) Reverse = reverse the passing order (e.g., 3 to 2, 2 to 1 etc.). (2) Switch= move your ball to the next group clockwise (3) Rotate = move your whole group to change position with another group.  ****  **Shooting Challenges:**  In pairs, players take a ball and hoop and find a safe space in the hall to practice the below levels in order. Once they have mastered the level successfully (i.e., consistent completion), they move on to the next. Each pair can be at a different level to the others within the group.   \| 1. **Shoot to land in partner’s hands \| ****2. Shoot over partner (arms by sides) into hoop 2m away \| ****3. As before but partner can now raise arms to block. \| ****4. Partner can now jump to block also. \| ****5. Both players now have hoops and play 1v1 game. \| **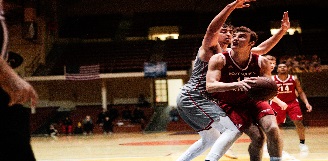**6. Players then use hoop to practice 1v1 shooting. \| \| --- \| --- \| --- \| --- \| --- \| --- \|   **Contingency game idea**  If participants find the paired shooting challenges too easy or prefer a group based game:  Shooting Alamo. Two queues start at either end of the court. One at a time players dribble up to a cone and attempt to shoot into the hoop (distance of cone from hoop can be adjusted to adapt challenge). Once a player has shot they retrieve their ball and join the opposite queue and wait to shoot down the opposite end. The players try to score as many times in a set period of time.  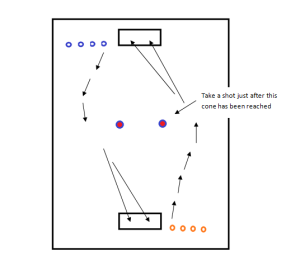 | - Run by 1 facilitator; others participate or engage with those on sideline - Can be creative and adaptive levels to make harder/easier. |
| (BREAK) | 5 min | To allow participants to catch their breath, get a drink or a snack, and engage in informal social interaction. Facilitators are to engage participants in casual conversation (water, electrolytes, snacks available). | | |
| PLAY | 30 min | - skill execution/ demonstration - teamwork/social interaction | **Zone ball**  Players are organised into two even teams on a regular basketball court with players spread across three separate zones (see figure xx). The aim is of the game is to score by throwing the ball through the hoop their team is attacking. The following rules are applied to allow players to adapt to the challenge:   1. Players cannot move with ball and must stay in zone (passing between each other only) 2. Players can now move three steps when in possession of the ball but must still remain in their zone. 3. Players can now move into the zone closest to them if they pass the ball into that zone. 4. Players can now move freely (no step limit) with their zone and with step 3 still applied.   Finally, players move into a regular basketball game and are able to move freely between all zones with no constraint on steps taken.  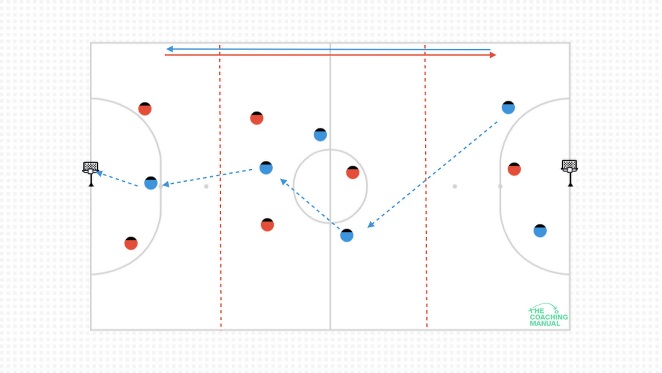  *To adjust the challenge the following adaptations were made where needed:  Struggling   - 3m safe zone = opposition players must stay 3 steps back from player in possession. - Add neutral player to support attacking team. The team with the ball will always therefore have an extra player. - Retreat rule. = opposition must move back to their half when ball is played from baseline.   Striving   - Three second rule = these players are only allowed to possess the ball for three seconds at a time. - Constrain advanced players to dribble with only less dominant hand. | - Run by 2 facilitators, other participates or engages with those on sideline - Provide opportunities for graded participation if necessary |
| (BREAK) | 3 min | To allow participants to catch their breath, get a drink or a snack, and engage in informal social interaction. Facilitators are to engage participants in casual conversation (water, electrolytes, snacks available). | | |
| COOL DOWN/DEBRIEF | 10 min | - lower heart rate - catch breath - stretch major muscle groups - begin habit of reflection | - Find someone to walk with to the other side of the court and back and share your favourite part of the session - Hamstring stretches- grass grazers (step forward while sweeping arms toward ground) - Reach arms up (deep breath in)/ reach to toes/knees/shins (breath out) x 3 - Repeat confidence meter and have participants compare to where they were at the beginning of session | Run by 1 facilitator; others to fetch food out of refrigerator and set up |
| SOCIAL TIME/ SNACKS | 15-20 min | - informal social interaction - rehydrate and refuel healthily | light snacks (i.e., wraps, sandwiches, fruit) and water/electrolytes available | - Ask participants to return watches and record steps taken - Distribute vouchers - Facilitators to engage young people in casual conversation |
| FACILITATOR REFLECTION | 30 min (post session) | - good, better, how approach - to promote reflection and begin to plan modifications for next week | - Good, better, how:   - RE: outcomes, logistical, design, overall (timing, progression, engagement, level, interaction, food, breaks, adverse events) - What went well? - What could be better and how? - How were the program components in relation to skill level? - How was the flow? - How was the timing? - How were the engagement levels? - How was interaction with the participants? - How did the team manage adverse events? - What modifications need to be made for next week? |  |

| **Session 2: Basketball (indoor)** | | | | |
| --- | --- | --- | --- | --- |
| *Key Session Outcomes:*1) Building rapport/building trust;  2) Social interaction; 3) Skill development / confidence; 4) Mental skills training: motivation; 5) Increased physical activity  *Materials needed*: basketballs (10-20), ball pump, cones (30), bibs (20), first aid kit, alternative activities (e.g., ring toss, skittles, beach bats), water, snacks, speaker  *Note:* Facilitators are to treat all participants and support workers equally; support workers and facilitators participate alongside young people for all activities | | | | |
| PHASE | Time | KEY COMPONENTS TO TARGET | ACTIVITIES | KEY TEACHING/ FACILITATING POINTS |
| INTRODUCTION | 15 min | - reminder of expectations - build comfort/familiarity with group and program - get to know names | **Arrival Activity**: skittles, beach bats, ring toss etc.; music  **Acknowledgement of Country:** same as session 1  **Welcome**   - Introductions Housekeeping reminders (toilets, snacks, breaks, etc) - recap expectations created as a group in week 1 (while adding basic stretches- calf raises; leg swings; lunges)   **Ice breaker**: get in order of birthdays; share birthday and name  **Life Skills**   - “Find your Why”   - intrinsic vs. extrinsic motivation discussion (present as red vs. green motivation)   - brainstorm reasons “why” people might play sport as group   - individuals to record their red vs. green “whys” for coming to program in workbook - Set up watches- guess how many steps? (record in workbook; closest from last week picks music) | - Engage participants in casual conversation upon arrival - Give participants watches and as they arrive (optional) - Encourage participants to engage in arrival activity (beach bats, ring toss, etc.) - Run by 1 facilitator, others participate |
| PHYSICAL WARM UP | 10-15 min | - elevated heart rate - movement in major muscle groups - social interaction/ familiarity - priming for physical skills required for day (e.g., hand-eye coordination, reflexes, spatial awareness) | **Dynamic movement**   - HSNT/Cone:(best ⅔): In pairs with 1 cone per pair. Facilitator calls out “head, shoulders, knees, cone” in random order. Participants are to touch different parts on their own body when called. When “cone” is called, participants compete to grab the cone first (challenge: turn around)   **Foxes and farmers tag** (see diagram)   - One participant starts as the catcher (farmer) - All other participants start as runners (foxes) - Foxes begin with a bib tucked into the back of their shorts - The farmer aims to run around and remove bibs from the foxes. - If a fox loses their bib they then become a farmer. - The last fox in the box is the winner.      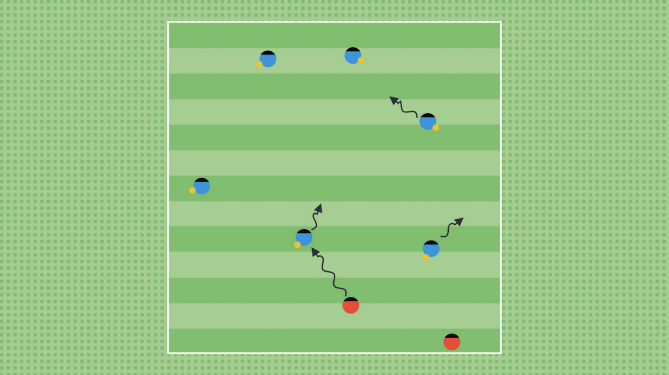 | - Run by 1 facilitator, others participate or engage with those on the sideline - In head, shoulders, knees, and cone- facilitator to walk around so everyone can hear - facilitators to explain why (in relation to basketball) during explanation (e.g., physical mobility, warming up major muscle groups, injury prevention, spatial awareness, reflexes, etc |
| (BREAK) | 2-3 min | To allow participants to catch their breath, get a drink or a snack, and engage in informal social interaction. Facilitators are to engage participants in casual conversation. | | |
| SKILL LEARNING | 20 min | - recap shooting/passing - dribbling - defending - social interaction - physical activity | **Confidence meter**: how is this different than last week? (create a line of cones going from red to yellow to green; have participants and facilitators line up according to their basketball confidence/experience- red being low confidence/experience; green being high).  **Skill development**   - 3 on 2 defense and attack (2 defenders vs 3 attackers; 1-2 in the rest zone; rotate after each score)   - challenge**:** 3 defenders vs. 2 attackers   - option: progress via wave practice to integrate whole group - Team Shoot off: 2 teams aiming to score from four balls against two defenders.   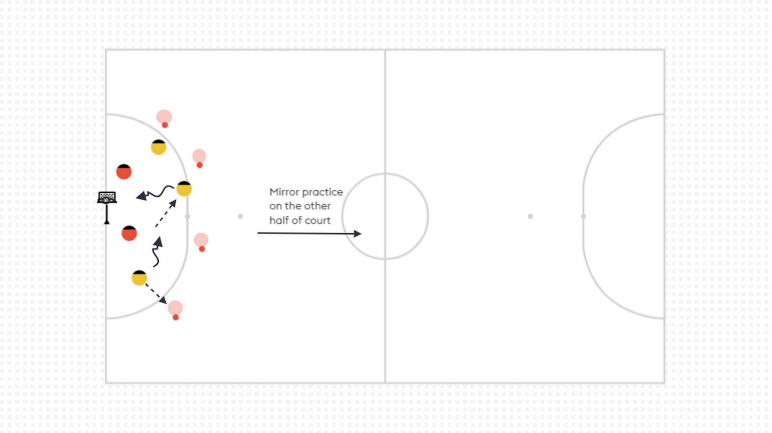   - Constraints (from attackers perspective)   - Struggling: Defenders must hold bib together whilst defending   - Striving: Must shoot within 15 seconds of picking ball up off the cone   - Progression: Can you score more points than the team in the opposite half | - 2 facilitators to deliver, 1 to participate or engage with those on the sideline - facilitators to encourage reciprocal learning and increased challenges in skill development - facilitators to take mental notes of participants helping/coaching/supporting one another - add water break in middle if needed - facilitators to prompt use of life skill (motivation) where appropriate |
| (BREAK) | 3 min | To allow participants to catch their breath, get a drink or a snack, and engage in informal social interaction. Facilitators are to engage participants in casual conversation. | | |
| PLAY | 30 min | - skill execution/demonstration - teamwork/social interaction - physical activity - opportunity to use life skill (“find your why”) | **Zone ball**  Players are organised into two even teams on a regular basketball court with players spread across three separate zones (see figure xx). The aim is of the game is to score by throwing the ball through the hoop their team is attacking. The following rules are applied to allow players to adapt to the challenge:   1. Players cannot move with ball and must stay in zone (passing between each other only) 2. Players can now move three steps when in possession of the ball but must still remain in their zone. 3. Players can now move into the zone closest to them if they pass the ball into that zone. 4. Players can now move freely (no step limit) with their zone and with step 3 still applied.   Finally, players move into a regular basketball game and are able to move freely between all zones with no constraint on steps taken.  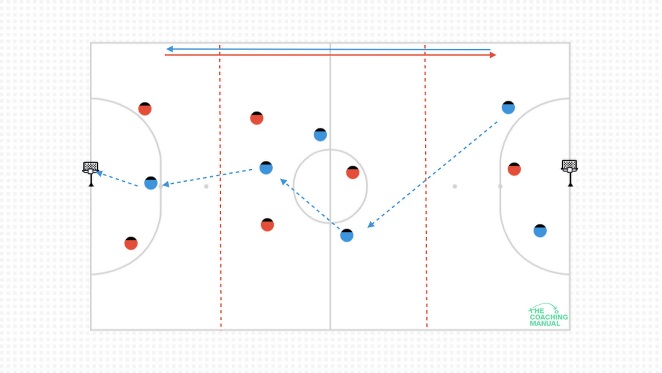  *To adjust the challenge the following adaptations were made where needed:  Struggling   - 3m safe zone = opposition players must stay 3 steps back from player in possession. - Add neutral player to support attacking team. The team with the ball will always therefore have an extra player. - Retreat rule. = opposition must move back to their half when ball is played from baseline.   Striving   - Three second rule = these players are only allowed to possess the ball for three seconds at a time. - Constrain advanced players to dribble with only less dominant hand. | - - delivered by 1 facilitator; 2 to participate or interact with those on the sideline   - be aware of bib sizing when determining teams   - be aware of skill level when assigning teams (strongest players on opposite teams)   - add water breaks throughout if needed   - facilitators to prompt use of life skill (motivation) where appropriate |
| COOL DOWN/DEBRIEF | 10 min | - lower heart rate - catch breath - social interaction - begin habit of reflection | **Cool down**   - walk with another: share one thing the other person did well and/or fav part of session? - Reach arms up (deep breath in)/ reach to toes/knees/shins (breath out) x 3 - confidence meter- compare to beginning of session   **Debrief**   - encourage participants to reflect on “find your why” for session in notebooks- what kept you motivated? - prompt “find your why” reflection/discussion for other areas in life- what is your motivation for other areas of life? How can you increase your “green” motivation?   **Vote**: next week’s sport: ultimate frisbee, touch rugby, soccer | one facilitator to deliver; two others to set up food etc. |
| SOCIAL TIME/ SNACKS | 20 min | - informal social interaction - rehydrate and refuel healthily | light snacks (i.e., wraps, sandwiches, fruit) and water/electrolytes available | - Ask participants to return watches and record steps taken - Distribute vouchers - Facilitators to engage young people in casual conversation |
| FACILITATOR REFLECTION | 30 min (post session) | (same as session 1) | (same as session 1) | |
|  |  |  |  |  |

| **Session 3: Touch Rugby (indoor)** | | | | |
| --- | --- | --- | --- | --- |
| *Key outcomes*: 1) Building rapport/building trust; 2) Social interaction; 3) Skill development / confidence; 4) Increased physical activity; 5) Mental skills training: goals  *Materials needed:* Equipment: rugby balls (10-20), cones (30),bibs (20), first aid kit, alternative activities (e.g., ring toss, skittles, beach bats), water, snacks  *Note:* Facilitators are to treat all participants and support workers equally; support workers and facilitators participate alongside young people for all activities | | | | |
| PHASE | TIMING | KEY COMPONENTS TO TARGET | ACTIVITIES | KEY TEACHING/ FACILITATING POINTS |
| INTRODUCTION/ ICE BREAKER(S) | 20 min | Build rapport and familiarity, foster social interaction, introduce mental skills component | **Arrival Activity**: skittles, beach bats, ring toss etc.; music  **Acknowledgement of Country:** same as session 1  **Welcome:** introductions, housekeeping reminder; recap expectations (while adding in basic stretches- calf raises; leg swings; lunges)  **Ice breaker:** find someone else; learn name and favourite meal to cook or have cooked for them; introduce partner to group  **Life skills**   - Check in: “Find your Why”- did you notice red/green motivation in your life? Connect motivation to goal setting - Set up watches (closest steps from last week picks music) - Action goals   - Something in the here and now that is measurable; important in sport training   - Includes What? How? and Why? components   - Have each person set an action goal; write in workbook and include what, how, and why elements. (ex: number of steps per session, participate in all activities, get to know someone new) | - Engage participants in casual conversation upon arrival - Give participants watches and as they arrive (optional) - Encourage participants to engage in arrival activity (beach bats, ring toss, etc.) - Run by 1 facilitator, others participate |
| PHYSICAL WARMUP | 10 min | - elevated heart rate - movement in major muscle groups - social interaction/familiarity - priming for physical skills required for day (e.g., hand-eye coordination, reflexes, spatial awareness) | **Over/under Relay**   - Participants start in queues of 5 to 7 with the person at the front of each queue starting on line 1 with a ball in their hands (see diagram) - On the ‘go’ the teams pass the ball between them in an ‘over-under’ fashion whereby the first person passes the ball over their head followed by the second person passing the ball between their legs and so on. - When the ball reaches the final person they run to the front of the queue one step ahead of the person behind them and begin the process again. - The aim is for the queues to race by progression their queue forwards in this manner towards the finish line (line 2 in diagram)   Progressions:   - - Alternate the challenge to ‘side to side’ (i.e., moving the ball around the left hand side of your body followed by the next person moving it around their right hand side)   - Combine a combination of over-under and side to side (i.e. “a over- under-side-side” pattern)   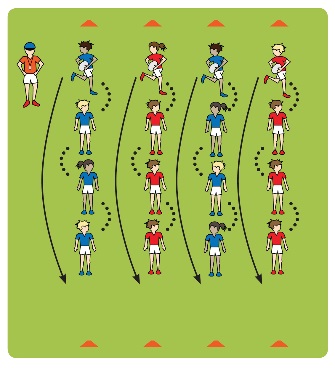  **Ball Tag**   - One participant starts at the tagger with a ball in their hands. - The rest are runners and find a space within a set, safe space (diagram 1) - The aim is for the tagger to run and touch another participant with the ball. - Once a player is tagged, they become the tagger and must now chase the other players. - The game comes to an end after a set period of time (e.g., 2 minutes)   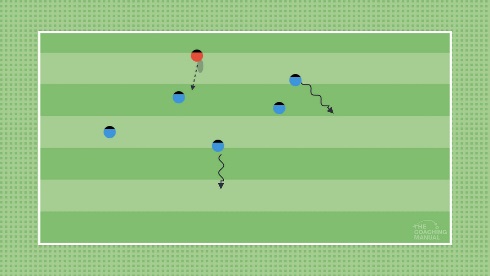  Progression   - Instead of the person being tagged switching with the tagger, they now join the tagger in catching the remaining runners. The ball may be passed between the taggers to tag runners. As more players are tagged, the more players the one ball can be passed between to catch the remaining runners.   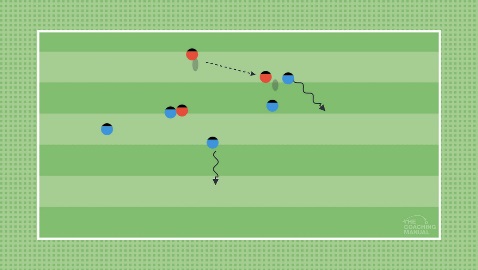  Contingency:   - For participants who don’t feel comfortable with the game environment individual ball handling challenges can be set. For example, Moving ball around body, Throw-clap-catch, figure of 8 (in and around legs) etc. | - Run by 1 facilitator, others participate or engage with those on the sideline - facilitators to explain why (in relation to rugby) during explanation (e.g., physical mobility, warming up major muscle groups, injury prevention, spatial awareness, reflexes, etc |
| (BREAK) | 5 min | To allow participants to catch their breath, get a drink or a snack, and engage in informal social interaction. Facilitators are to engage participants in casual conversation. | | |
| SKILL LEARNING | 20 min | - comfort with rugby ball, rugby rules familiarity, passing, spatial awareness, | **Confidence meter**: 1) touch rugby confidence; 2) confidence in ability to give it a go  **Wave passing drill**   - Participants start in groups of 4 to 6 standing side by side along a starting line (see diagram x.x) - On the command they aim to pass the ball along their line whilst running across to the finish line as a group - During this they must adhere to simple rules including:   - - 1. Passing the ball either sideways or backwards       2. Passing and receiving whilst moving in a forwards direction       3. Everyone must make or a receive a pass on each run through - Once the group has finished, the next group waiting at the finish line begins in the opposite direction (i.e., Wave 2).   Progressions   - Challenges are added that included the following   - Try to make as many passes as possible on each run (go all the way along and back)   - Try to add in overlaps/underlaps between players, i.e. moving in directions other than just straight lines.   - Add in static defenders who can intercept.   - Add in moving defenders who can intercept the ball.   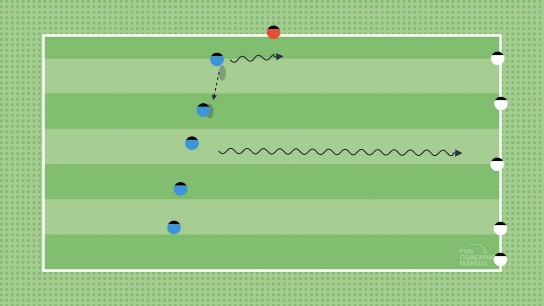  **Contingency activity:**  **“Catch this”**  As with basketball activity, players are each numbered from 1 to *n* within small groups (e.g., 4-6 players). Players start by passing in order (i.e., 1 to 2, 2 to 3, etc.) Players are then instructed to move ball around in a chest pass, bounce pass, and overarm passing techniques.  Progression: Instructions are then called out that all groups follow while still passing. (1) Reverse = reverse the passing order (e.g., 3 to 2, 2 to 1 etc.). (2) Switch= move your ball to the next group clockwise (3) Rotate = move your whole group to change position with another group.  Add in challenges such as:   - - Increase speed.   - Add in more balls   - Move as a group while passing the ball.   **** | - 2 facilitators to deliver, 1 to participate or engage with those on the sideline - facilitators to encourage reciprocal learning and increased challenges in skill development - facilitators to take mental notes of participants helping/coaching/supporting one another - add water break in middle if needed - facilitators to prompt use of life skill (action goals) where appropriate |
| (BREAK) | 5 min | To allow participants to catch their breath, get a drink or a snack, and engage in informal social interaction. Facilitators are to engage participants in casual conversation. | | |
| PLAY | 30 min | - skill execution/demonstration - teamwork/social interaction | **Constraints based approach to tag rugby game**  Set up (see diagram):   - A pitch is marked out with four boxes in each corner - Two even teams are created.   Rules:   - Each team tries to score by running over end line with the ball (Not diving touchdown) - Constraints (Progressed in order to allow the game to progress from simple to more complex rules  1. Rugby netball. Players cannot move with the ball and can only intercept to win possession. 2. Players get three steps when they have ball in hands. 3. Players can move freely with the ball but must pass (free pass) to teammate if they are tagged by opposition. 4. Pass they make following being tagged must now move backwards only. 5. All passes must now move backwards. (Normal tag ruby rules)   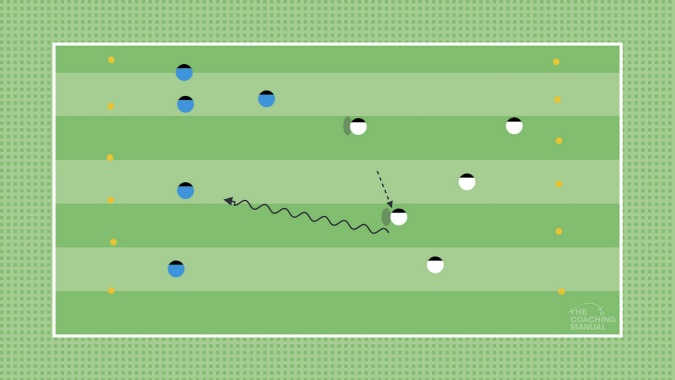 | - delivered by 1 facilitator; 2 to participate or interact with those on the sideline - be aware of bib sizing when determining teams - be aware of skill level when assigning teams (strongest players on opposite teams) - add water breaks throughout if needed - facilitators to prompt use of life skill (motivation) where appropriate - facilitators to explain where/how to “tag”; reinforce that it is tag and not contact |
| COOL DOWN/  DEBRIEF | 15 min | - lower heart rate - catch breath - promote habit of reflection | **Cooldown**   - Walk with someone- share favourite part of session - Hamstring stretch- “grass grazers” - Glute stretch- figure 4 or foot to bum - Reach arms up (deep breath in)/ reach to toes/knees/shins (breath out) x 3   **Debrief**   - **confidence meter:** 1: touch rugby; 2: ability to give it a go next week (prompt comparison to beginning of session - check in with goal… was it realistic? Too hard? too easy? what helped you accomplish it? What would help you accomplish next week? - Discussion/workbooks   - have participants apply action goal concepts to other goals they are currently working on (one thing that you can do today, tomorrow, or next week?; how, why?)   - Encourage writing in notebooks and sharing with person next to them and/or group | Collect watches and HR monitors, record steps, distribute vouchers |
| SOCIAL TIME/ SNACKS | 20 min | - informal social interaction - rehydrate and refuel healthily | light snacks (i.e., wraps, sandwiches, fruit) and water/electrolytes available | - informal social interaction - rehydrate and refuel healthily |
| FACILITATOR REFLECTION | 30 min (post session) | (same as session 1) | (same as session 1) | (same as session 1) |

| **Session 4: Touch Rugby (outdoor)**  *Key Outcomes:* 1) Building rapport/building trust; 2) Social interaction; 3) Skill development / confidence; 4) Increased physical activity; 5) Mental skills training: breath control; 6) cater to individuals (e.g., injury, personal goals)  *Materials needed*: rugby balls (10-20), cones (30),bibs (20), first aid kit, alternative activities (e.g., ring toss, skittles, beach bats), water, snacks  *Note:* Facilitators are to treat all participants and support workers equally; support workers and facilitators participate alongside young people for all activities | | | | |
| --- | --- | --- | --- | --- |
| PHASE | TIMING | KEY COMPONENTS TO TARGET | ACTIVITIES | KEY TEACHING/ FACILITATING POINTS |
| INTRODUCTION/ ICE BREAKER(S) | 20 min | Build rapport and familiarity, foster social interaction, introduce life skills component | **Arrival Activity**: skittles, beach bats, ring toss etc.; music  **Acknowledgement of Country:** same as session 1  **Welcome:** introductions, housekeeping reminder; recap expectations (while adding in basic stretches- calf raises; leg swings; lunges)  **Ice breaker:** find a partner and share an action goal incorporated into your week last week, or one that you can incorporate this week  **Life skills**   - breathing activity: “circle breath”   - Complete breath- slow and controlled   - Start with one hand on stomach and one hand on chest   - Think about lungs expanding from bottom/diaphragm   - Inhale through nose, out through mouth, pausing between   - Exhale should be longer than the inhale (ie in for 4, out for 5- modify for each individual) - Have participants repeat exercise; have those using a HR monitor note their HR before and after exercise, and to note resting HR mentally or in workbook - Discuss the importance of breath control in sport | - Engage participants in casual conversation upon arrival - Give participants watches and HR monitors and as they arrive (optional) - Encourage participants to engage in arrival activity (beach bats, ring toss, etc.) - Run by 1 facilitator, others participate |
| PHYSICAL WARMUP | 10 min | - elevated heart rate - movement in major muscle groups - social interaction/familiarity   priming for physical skills required for day (e.g., hand-eye coordination, reflexes, spatial awareness) | **Dynamic movement: Naughts and crosses**   - Create large 3x3 grid with cone - Teams compete to get 3 bibs (each team has a different colour) in a row. - Teams compete simultaneously (not turn by turn) - Players on each team take turns running the bib; must return to team tag next player relay style before they run the bib - Adaptations   - To make easier/encourage leadership, Add in guidance player at the end of the box to help.   - Challenge: can remove other team’s bib instead of adding your own   **Tag Game: mice tails**   - Players run from end to end without getting their bibs removed by taggers in the middle. - Players must hold a ball each - If tagged (bib removed) they become a tagger in the middle. - Last one crossing is the winner.   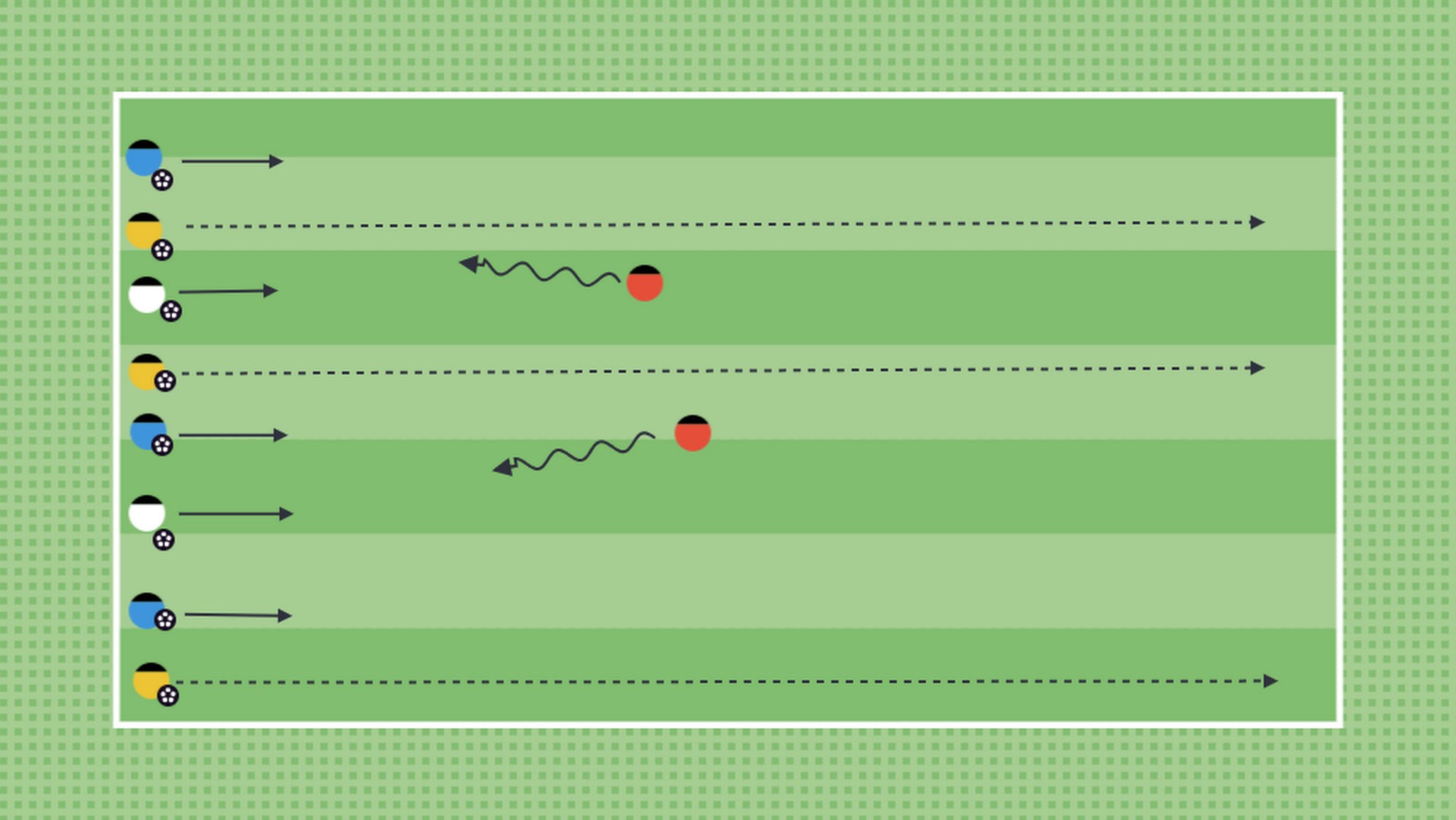 | - Run by 1 facilitator, others participate or engage with those on the sideline - facilitators to explain why (in relation to rugby) during explanation (e.g., physical mobility, warming up major muscle groups, injury prevention, spatial awareness, reflexes, etc - Prompt use of circle breathing where appropriate |
| (BREAK) | 5 min | To allow participants to catch their breath, get a drink or a snack, and engage in informal social interaction. Facilitators are to engage participants in casual conversation.  **Note:** prompt use of circle breathing during breaks, and have participants take note of HR monitor before and after break/breath work | | |
| SKILL LEARNING | 20 min | comfort with rugby ball, rugby rules familiarity, passing, spatial awareness | **Confidence meter**: 1: touch rugby; 2: ability to give it a go (compare to last week)  **‘Retrieve the ball’**  Set up:   - - Two teams of approx. 6-8 members pass three balls around within a predefined area (see diagram 1).   - The three balls each have a different sign marked on them **O, X,** 🔺.   Stage 1   - - When the coach calls the sign the teams must work out where this ball is and pass to the coach before the other team. - Stage 2   - A defender is added to each box from the opposite team.   - On the call of a certain sign, the defender aims to retrieve the by tagging players in possession of it. On tagging the player they inspect the ball. If they get the incorrect ball they must pass this back to the team (remembering where it was) and try again to regain correct ball. Whichever defender retrieves the correct ball first wins a point for their team. - Stage 3   - The coach now calls all three signs in a specific order.   - The defender must now retrieve all three balls in the correct order in the same manner of tagging players. e.g., **X then** 🔺 **then O.**   - Again, the defender that retrieves the balls fastest wins one point for their team.   - The game is over either after a predetermined period of time or certain score is attained.   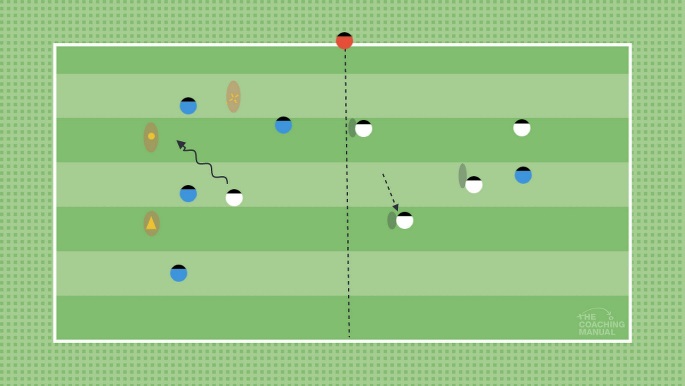 | - facilitators to create/seek opportunities to foster individual needs (e.g., catering to injury; providing opportunity for leadership) - 2 facilitators to deliver, 1 to participate or engage with those on the sideline - facilitators to encourage reciprocal learning and increased challenges in skill development - add water break in middle if needed - facilitators to prompt use of life skill (circle breath) where appropriate |
| (BREAK) | 5 min | To allow participants to catch their breath, get a drink or a snack, and engage in informal social interaction. Facilitators are to engage participants in casual conversation.  **Note:** prompt use of circle breathing during breaks, and have participants take note of HR monitor before and after break/breath work | | |
| PLAY | 30 min | - skill execution/demonstration - teamwork/social interaction | **4 corner ball**  Set up (see diagram):   - A pitch is marked out with four boxes in each corner - Two even teams are created.   Rules:   - The ball can move in any direction and players can also run with the ball in their hands. - Any team can score in any corner - Once a player is tagged they must stop and pass the ball to a teammate. - After a team has received 3 tags they must turn the ball over to the opposition. - Players can only be tagged when in possession of ball   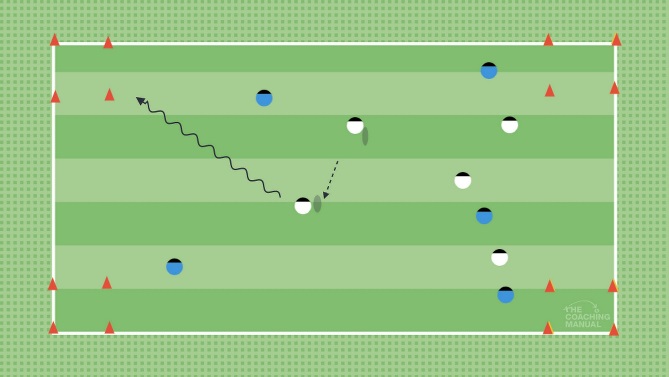  Progression   - Players can only score by receiving a pass in one of the four boxes (scoring team keeps possession; one free pass after scoring or turnover) - Players can only score by receiving a pass in one of the four boxes. However, on scoring that team must switch directions and try to score in other end to confirm the goal - Coaches set specific ends that each team must score (see diagram 2). Player must touch ball on ground to score. Players can run in or be passed to in either or their target boxes.   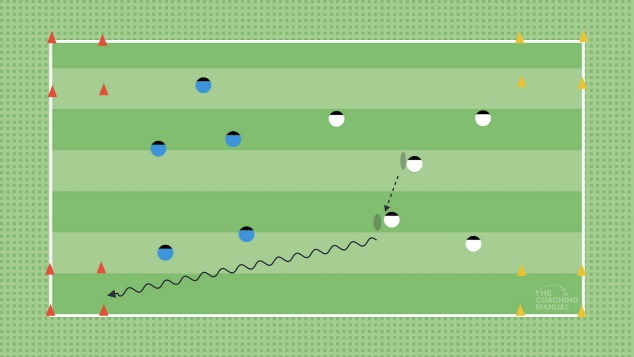  Progression rules   1. Ball can now only move backwards between teammates. 2. Players attack one end zone replicating a conventional rugby pitch as opposed to original target boxes.   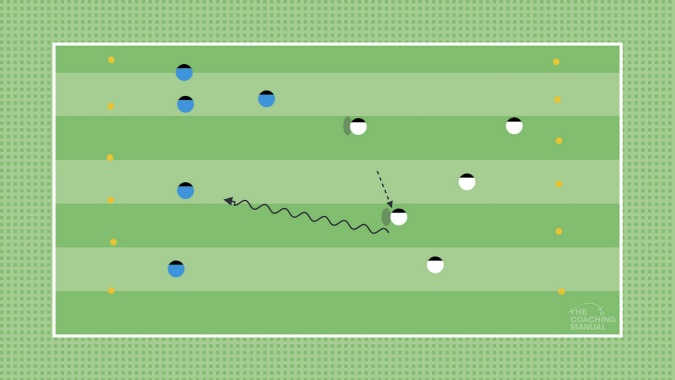 | - delivered by 1 facilitator; 2 to participate or interact with those on the sideline - facilitators to explain where/how to “tag”; reinforce that it is tag and not contact - facilitators to determine result of dropped ball or missed pass depending on skill levels (e.g., play on, or turnover) - facilitators to remind how/where to tag - be aware of bib sizing when determining teams - be aware of skill level when assigning teams (strongest players on opposite teams) - add water breaks throughout if needed - facilitators to prompt use of life skill (circle breath) where appropriate |
| COOL DOWN/DEBRIEF | 15 min | - lower heart rate - catch breath - develop habit of reflection | **Circle breath**: prompt use of circle breathing, and have participants take note of HR monitor before and after break/breath work  **Confidence meter:** touch rugby confidence (compare to beginning of session)  **Cooldown**   - Walk with someone- share favourite part of session - Hamstring stretch- “grass grazers” - Glute stretch- figure 4 or foot to bum - Reach arms up (deep breath in)/ reach to toes/knees/shins (breath out) x 3 - breath work paired with HR monitor   **Debrief**   - Notebooks: reflect on when/where would breath work be helpful - conversation about elevated steps and elevated HR in relation to health - **Vote on next week’s sport :** ultimate frisbee, soccer, hockey | - 1 facilitator to deliver; others to set up food etc. - Collect watches and HR monitors, record steps, distribute vouchers |

| SOCIAL TIME/ SNACKS | 20 min | - informal social interaction - rehydrate and refuel healthily | light snacks (i.e., wraps, sandwiches, fruit) and water/electrolytes available | - informal social interaction - rehydrate and refuel healthily |
| --- | --- | --- | --- | --- |
| FACILITATOR REFLECTION | 30 min (post session) | (same as session 1) | (same as session 1) | (same as session 1) |

| **Session 5: Hockey**  *Key outcomes:* 1. Building rapport/building trust 2. Social interaction 3. Skill development / confidence 4. Increased physical activity 5. Mental skills training: breath control  *Materials Needed:* hockey sticks (20), balls (20), cones (30),bibs (20), first aid kit, alternative activities (e.g., ring toss, skittles, beach bats), water, snacks  *Note:* Facilitators are to treat all participants and support workers equally; support workers and facilitators participate alongside young people for all activities | | | | |
| --- | --- | --- | --- | --- |
| PHASE | TIMING | KEY COMPONENTS TO TARGET | ACTIVITIES | KEY TEACHING/ FACILITATING POINTS |
| INTRODUCTION/ ICE BREAKER(S) | 20 min | Build rapport and familiarity, foster social interaction, introduce mental skills component | **Arrival Activity**: skittles, beach bats, ring toss etc.; music  **Acknowledgement of Country:** same as session 1  **Welcome:** introductions, housekeeping reminder; recap expectations (while adding in basic stretches- calf raises; leg swings; lunges)  **Ice breaker:** paper airplane/questions   - Provide each person with a piece of paper and a pen - Each person writes 1-2 questions they might want to know about others in the group (e.g., if you could travel anywhere in the world, where would it be and why?) - Each person folds paper into a paper airplane - At the same time, everyone throws paper airplane - Progress by picking up someone else’s paper airplane and throwing it (continue for a minute) - When the facilitator calls time, pick up a nearby paper airplane, find a partner, unfold paper airplanes, and ask and answer the questions on the paper with one another   **Life skills**   - breathing activity-review circle breath; begin with pushing all air out and holding to demonstrate effect; add pauses at top and bottom; challenge: extend count from last week - notice heart rate before and after practice - confidence meter (hockey ability; ability to give it a go) | - Engage participants in casual conversation upon arrival - Give participants watches and HR monitors and as they arrive (optional) - Encourage participants to engage in arrival activity (beach bats, ring toss, etc.) - Run by 1 facilitator, others participate |
| PHYSICAL WARM UP | 10 min | - elevated heart rate - movement in major muscle groups - social interaction/familiarity - priming for physical skills required for day (e.g., hand-eye coordination, reflexes, spatial awareness) | **Dynamic movement: Group bib tag**   - start slow to warm up (e.g., walking only) - progression - once tagged you go pick up a spare bib, work in teams with a bib - provide rest zone area - progress with sticks and balls: Catchers aim to throw bib on the balls of the runners. If caught a player is stuck until another runner passes their ball between their legs and releases them. | - 2 facilitators to deliver, 1 to participate or engage with those on the sideline - facilitators to create/seek opportunities to foster individual needs (e.g., catering to injury; providing opportunity for leadership) - facilitators to encourage reciprocal learning and increased challenges in skill development - add water break in middle if needed - facilitators to prompt use of life skill (circle breath) where appropriate - Use rest zone and constraints to encourage gradual warm up |
| (BREAK) | 5 min | To allow participants to catch their breath, get a drink or a snack, and engage in informal social interaction. Facilitators are to engage participants in casual conversation.  **Note:** prompt use of circle breathing during breaks, and have participants take note of HR monitor before and after break/breath work | | |
| SKILL LEARNING | 20 min | comfort with hockey stick, rules familiarity, passing, spatial awareness, | **“Bringing home the bacon”**  Set up:   - Two even teams are created with each team being numbered 1 to *N* (e.g., 1 to 10 if 10 players). - Within this set up each player has a player on the opposite team with the same corresponding number. Player are attempted to be matched on general ability. - A regular hocket pitch is create with both teams lined up along one sideline (see diagram 1) - A hockey ball is placed in the middle of the pitch.   Rules   - When the coach calls a number, the player with that number from each team runs out onto the pitch and attempts to score with the ball left in the middle of the pitch by striking it into the opposing team’s goal - If a player scores a goal this is added to their team total. - The game ends after a certain period of time or number of goals.   Progressions   - Coach may call 2 numbers with multiple players working together (e.g., passing, dribbling) to try and score a goal in line with the same rules - The coach can continue to increase the number of numbers call to enhance the difficult of the task.   Constraint options:   - If obvious differences in ability occur between two players with the same number the following constrains are imposed on the stronger player. - Stronger player can walk only - One handed hockey sticks - Player must balance cone on head while playing.   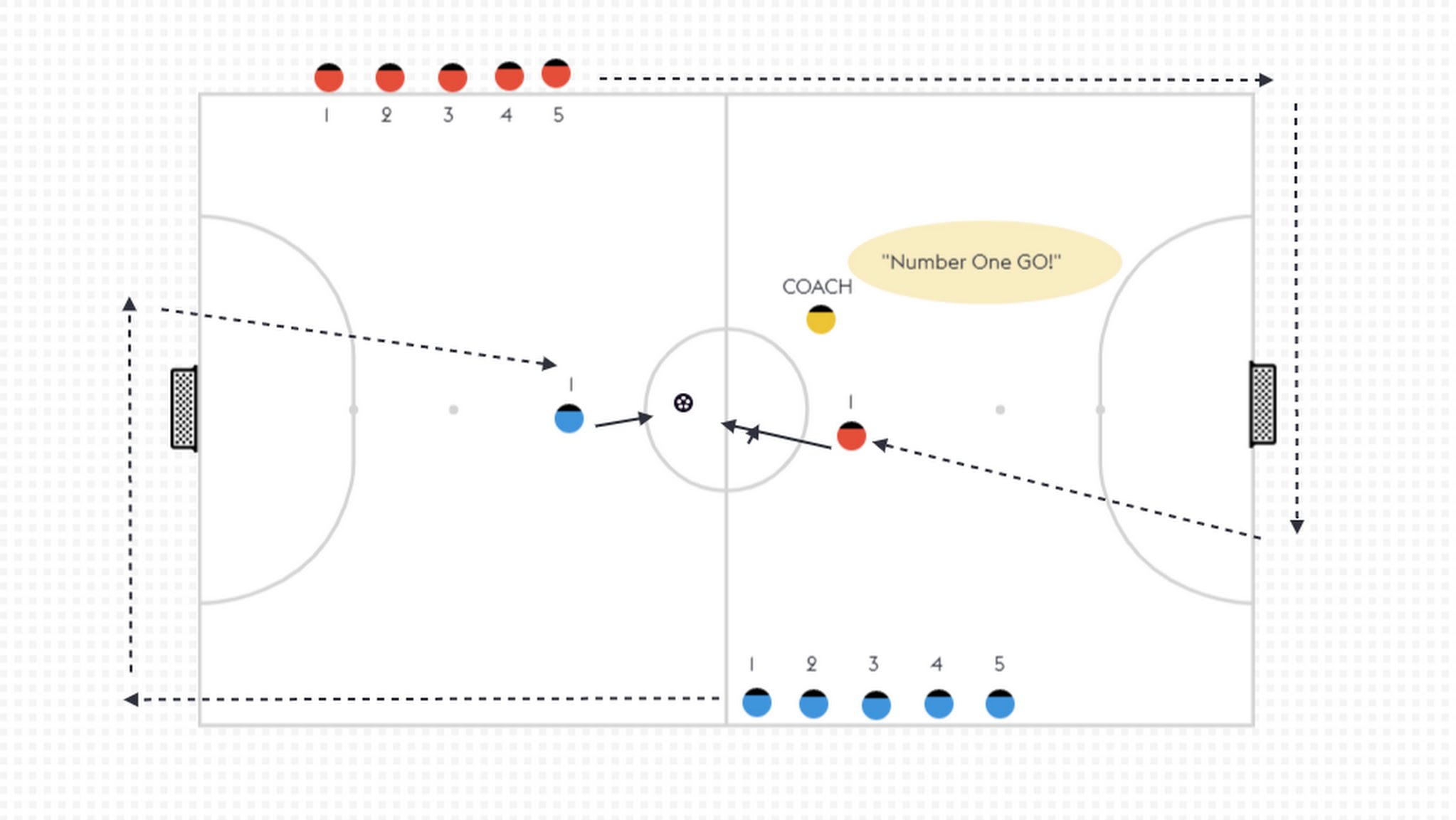 | adjust according to skill level and engagement |
| (BREAK) | 5 min | To allow participants to catch their breath, get a drink or a snack, and engage in informal social interaction. Facilitators are to engage participants in casual conversation.  **Note:** prompt use of circle breathing during breaks, and have participants take note of HR monitor before and after break/breath work | | |
| PLAY | 30 min | - skill execution/demonstration - teamwork/social interaction | Small sided game with above constraints if needed   - additional constraints   - safe zone   - neutral player   - round robin competition |  |
| COOL DOWN/DEBRIEF | 15 min | - lower heart rate - catch breath - foster habit of reflection | **Circle breath**: prompt use of circle breathing, and have participants take note of HR monitor before and after break/breath work  **Confidence meter:** touch rugby confidence (compare to beginning of session)  **Cooldown**   - Walk with someone- share favourite part of session - Hamstring stretch- “grass grazers” - Glute stretch- figure 4 or foot to bum - Reach arms up (deep breath in)/ reach to toes/knees/shins (breath out) x 3 - breath work paired with HR monitor   - Rhythmic breathing: inhale for 4, hold for 4, exhale for 4, pause for 4.   **Debrief**   - Notebooks: reflect when/where would breath work be helpful - Set action goal re: breath work: what, how, why | - 1 facilitator to deliver; others to set up food etc. - Collect watches and HR monitors, record steps, distribute vouchers |
| SOCIAL TIME/ SNACKS | 20 min | - informal social interaction - rehydrate and refuel healthily | light snacks (i.e., wraps, sandwiches, fruit) and water/electrolytes available | - informal social interaction - rehydrate and refuel healthily |
| FACILITATOR REFLECTION | 30 min (post session) | (same as session 1) | (same as session 1) | (same as session 1) |

| **Session 6: Hockey**  *Key outcomes:* 1. Building team cohesion 2. Social interaction 3. Skill development / confidence 4. Increased physical activity 5. Mental skills training: breath control 6. reflection  *Materials Needed:* hockey sticks (20), balls (20), cones (30),bibs (20), first aid kit, alternative activities (e.g., ring toss, skittles, beach bats), cardboard pieces for team building (15), water, snacks  *Note:* Facilitators are to treat all participants and support workers equally; support workers and facilitators participate alongside young people for all activities | | | | |
| --- | --- | --- | --- | --- |
| PHASE | TIMING | KEY COMPONENTS TO TARGET | ACTIVITIES | KEY TEACHING/ FACILITATING POINTS |
| INTRODUCTION/ ICE BREAKER(S) | 10 min | Build rapport and familiarity, foster social interaction, introduce life skills component | **Arrival Activity**: skittles, beach bats, ring toss etc.; music  **Acknowledgement of Country:** same as session 1  **Welcome:** introductions, housekeeping reminder; recap expectations (while adding in basic stretches- calf raises; leg swings; lunges)  **Life skills**   - Acknowledge last session. - Remind participants of “finding their why”- what brought you here today? What is one thing you want to get out of this session? (Action goal related to this: what, how, why) | - Run by 1 facilitator, others participate - Engage participants in casual conversation upon arrival - Give participants watches and HR monitors and as they arrive (optional) - Encourage participants to engage in arrival activity (beach bats, ring toss, etc.) |
| PHYSICAL WARM UP | 20 min | - movement in major muscle groups - social interaction/familiarity - team building - leadership and decision making opportunities | **Crocodile river**   - 2 teams trying to get across crocodile infested river - Can use “magic stones” (cardboard pieces) that float and keep you safe as long as there is bodily contact on them - As soon as no one is touching magic stones, they disappear - People are only safe on the stones- if a hand or foot touches the river, it will get bitten off and they must proceed without using it - If someone ends up in the river with no part of them on the stone, the whole team has to start over - Play - one stone for each person on the team - river as wide as 1.5 meters x people on team - teams cross in opposite directions - give 2-3 minutes to strategise/practise before starting - goal is to be the first team with all members safely across - Challenge: add 1 cone per team as “oxygen mask” that everyone must breath into once every minute to stay alive   **Zip tag (**If time allows and/or not liking problem solving)  Set up:   - Players all start in the box with. - Taggers tag by brushing the back of runners with their hand (i.e., pulling down an imaginary zip on runners backs) - If a player is tagged the must crouch down until they are ‘zipped’ back up by someone brushing their hand up on their back. - The game is over when there are no more runners remaining or after a predestined period of time.   Stage 1:   - Three taggers are placed in the box. - Everyone else is a runner. - Any runner can zip a caught person back up and bring them back into the game.   Stage 2:   - Three medics are added to the game, keeping the three taggers. - In this stage, only medics can zip players back up making it harder to keep runners in the game. - Note. Medics cannot be unzipped at this point.   Stage 3:   - Players are grouped and bibbed in four equal teams - In this version players can tag players from any of the other three teams. - However, only your teammates can re-zip you and bring you back into the game if you are caught. - Once your team is all zipped your team is out of the game. | - 1 facilitator to lead, 2 others to moderate - be aware of even mix of participants on teams; encourage opportunities to lead |
| (BREAK) | 5 min | To allow participants to catch their breath, get a drink or a snack, and engage in informal social interaction. Facilitators are to engage participants in casual conversation.  **Note:** prompt use of circle breathing during breaks, and have participants take note of HR monitor before and after break/breath work | | |
| SKILL LEARNING | 20 min  2:30-2:50 | comfort with sticks and balls, hockey rules familiarity, passing, tackling, spatial awareness | **Guardians of the Galaxy**  Set up:   - A box is created with 4 different colour gates (two cones of the same colour) placed at different points within. - At least 3 sets of each colour gates are needed - Players are then put in pairs each with a stick and one ball between them   Rules   - The aim for the players is to pass the balls between as many gates as possible. - Players take turn to dribble the ball in between passing through each set of gates. - Player can’t pass through the same colour twice in a row - The aim for pairs is to try to pass through as many as gates as possible in a set period of time.   Progressions:   - Defenders are added in the box to try and tack the players dribbling and knock their ball out of the box. - If their ball leaves the box, a pair’s score reverts back to zero.   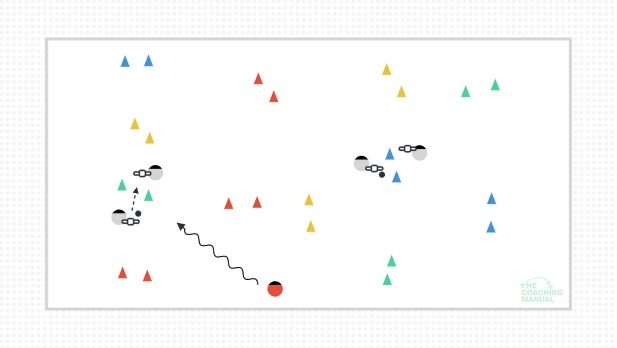  **Risky business**  Set up   - Two teams are created each with an even number of players. - Each team takes turn as the attacking team.   Rules   - As the attacking team, each individual in turn gets to choose a challenge for their team to score a certain amount of points. - Points are only attained if their team scores and the number of points give is progressive in line with the difficulty (see ‘options’ below). - 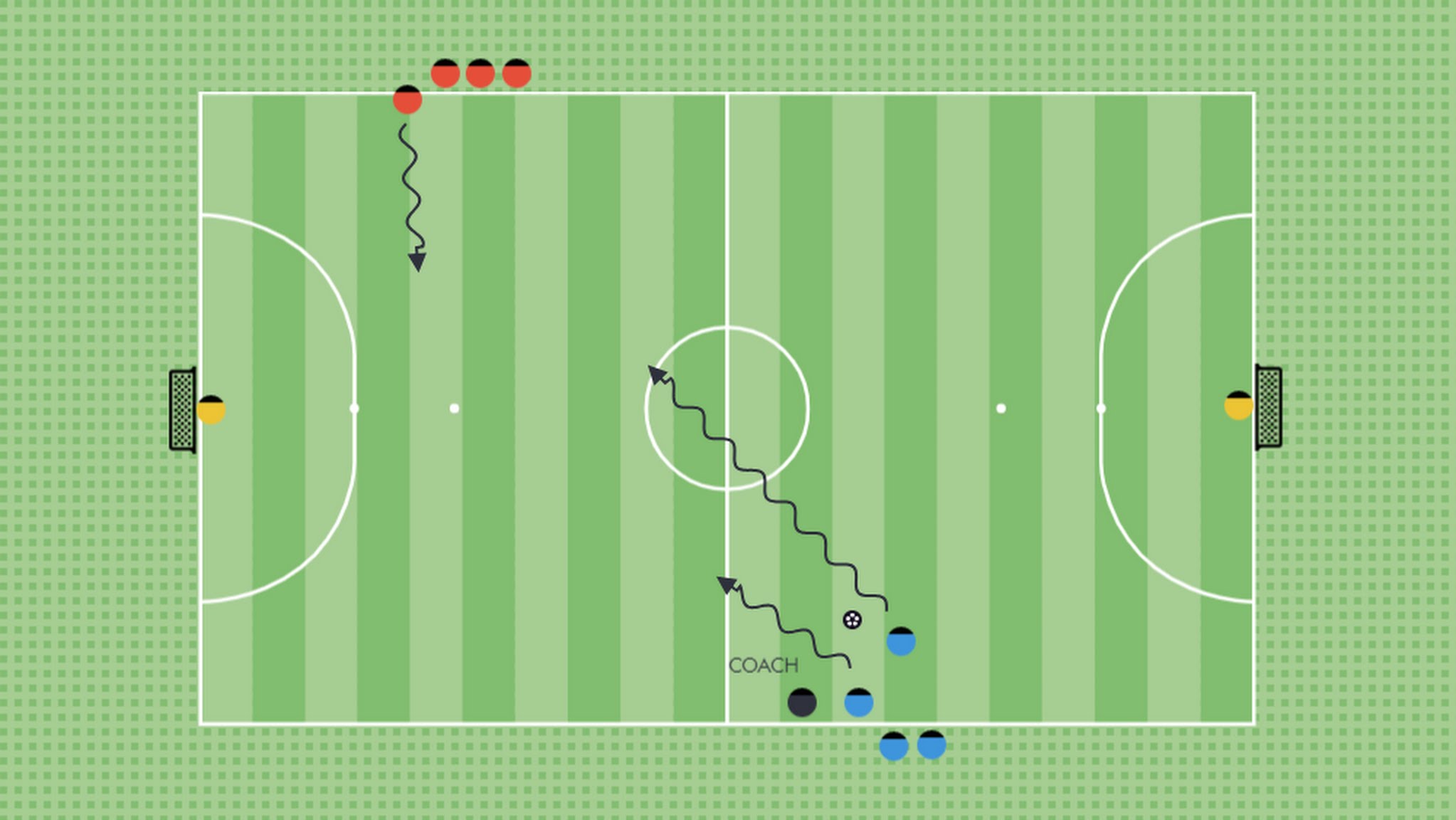Once the player chooses the challenge the coach plays the ball in and the players attempt to score past their defending opposition. - Once every player has chosen a challenge the other turn become the attackers and attempt to beat the opposition’s overall score to win the game. - Each team gets two attempts as the attacking team before scores are totalled and the winner determined. - Point Options   *5 Points = 1 attacker v 2 defenders*  *4 Points = 1v1*  *3 Points = 3v2*  *2 Points = 2v1*  *1 Point = 3v1* | - 2 facilitators to deliver, 1 to participate or engage with those on the sideline - facilitators to create/seek opportunities to foster individual needs (e.g., catering to injury; providing opportunity for leadership) - facilitators to take note of any adaptations needed/utilised - facilitators to encourage reciprocal learning and increased challenges in skill development - add water break in middle if needed - facilitators to seek opportunities to embed life skills training throughout |
| (BREAK) | 5 min | To allow participants to catch their breath, get a drink or a snack, and engage in informal social interaction. Facilitators are to engage participants in casual conversation.  **Note:** prompt use of circle breathing during breaks, and have participants take note of HR monitor before and after break/breath work | | |
| PLAY | 30 min | - skill execution/ demonstration - teamwork/social interaction | **Small sided game** with constraints if needed   - constraint options: - walking only - one handed hockey sticks - keep cone on head - additional constraints   - safe zone   - neutral player   - round robin competition   **Wrap activity**   - Penalty shootout | - facilitators to create/seek opportunities to foster individual needs (e.g., catering to injury; providing opportunity for leadership) - facilitators to take note of any adaptations needed/utilised - facilitators to seek opportunities to embed life skills training throughout |
| COOL DOWN/DEBRIEF | 20 min | - lower heart rate - catch breath - foster habit of reflection | **Cool down**   - Walk with someone- share favourite part of program - Hamstring stretch- “grass grazers” - Glute stretch- figure 4 or foot to bum - Reach arms up (deep breath in)/ reach to toes/knees/shins (breath out) x 3   **Debrief**   - think back to first week: confidence in ability to participate in program vs. today - Reflection in workbooks:   - What challenges did you overcome?   - What are you most proud of?   - What motivated you to attend?   - How did you ask for/receive/give support?   - What will you take forward from here? (set action goal) - Visualise transfer   - Hand out multi coloured post-it notes   - Have everyone answer: What will you take from this program to other parts of your life? (can write on multiple post-its)   - Instruct (and facilitators model) sticking post-its to board |  |
| SOCIAL TIME/ SNACKS | 20 min | - informal social interaction - rehydrate and refuel healthily | light snacks (i.e., wraps, sandwiches, fruit) and water/electrolytes available |  |
| FACILITATOR REFLECTION | 30 min (post session) | (same as session 1) | - (same as session 1) | (same as session 1) |
