# Supplementary material for: Enhancing functional recovery for young people recovering from first episode psychosis via sport-based life skills training: outcomes of a feasibility and pilot study
Source: Health Psychol Behav Med. 2022 Nov 21;10(1):1136–58. doi: 10.1080/21642850.2022.2147073 (PMC9683043; doi:10.1080/21642850.2022.2147073)
Supplement: Supplemental Material [file RHPB_A_2147073_SM7104.zip › HPBM-2022-0007.R2_supp_tables.docx]

Table S1

*Session Feedback, Reflections and Modifications*

| **Session # and notes** | **Facilitator Reflection** | **Young Person Feedback** | **Support Worker Feedback** | **Modifications** |
| --- | --- | --- | --- | --- |
| - **Session 1** - Indoor basketball - 4/5 young people attended - 7 support workers attended (2 peer support workers) | **What went well:**   - Initial rapport building went well; it was helpful to engage with young people as they came in and during the breaks. - Engagement and buy in was high- everyone participated the whole time. Young people appeared more comfortable as the sessions progressed - The progression appeared to work well to help participants feel comfortable and build some rapport and group cohesions. It seemed helpful to allow the young people to work in pairs with their support worker at first before progressing to smaller groups and then to a large group activity. - The warm up games in pairs (e.g., head, shoulders, knees, and toes) and then in larger groups (e.g., bib tag) appeared to really help break the ice- participants were laughing and connecting after this - By the end of the session, participants appeared to be more confident and comfortable. This was expecially evident after, for example, moments of celebration (e.g., fist pumping, yelling out) after scoring a basket - It was interesting to see the young people helping their support worker or one another with some of the skill learning (e.g., instruction on how to shoot properly) - Constraints (e.g. playing just in your zone at first) appeared to be helpful in increasing buy in for those with limited basketball experience - Support workers modelled participation well | **What went well:**  Phone interview with young person:   - Favourite part of session was the warmup; helped the young person learn to socialise and get more comfortable with others - Enjoyed the food and the electrolytes- wasn’t expecting that - Was proud to have overcome challenges of socialising and engaging in PA for the whole session - The social skills were most useful to apply to other parts of life - Really enjoyed that it was a fun, non-competitive environment; got to use prior sport skills, but in a fun way - Reported that “headspace felt great after”; felt “chirpy” and happy - Was surprised to feel so comfortable around the facilitators; enjoyed that they came up to chat, laughed at themselves, and created an easy going environment. Young person reported feeling very accepted, and much more open than expected - Will attend again (10/10)   Phone interview with young person   - Enjoyed whole session - Favourite part was the large group basketball game and the “chilled” time at the end - Appreciated the food and the electrolytes - Will attend again (10/10)   Email response from young person:   - “I had a great time at the program” - “My favourite part was playing a game at the end and I also enjoyed the ball catching drills we did for skills” - “I think you did a good job supporting me” | **What went well:**  Community worker phone interview (primary support worker of 2 participants):   - Observed levels of engagement, enjoyment, openness, and confidence in clients not seen before - Enjoyed seeing clients helping other young people with shooting skills - Enjoyed seeing young people helping support workers with skills and with rules knowledge - Expressed that young people demonstrating helping behaviours could be empowering for them and helpful for recovery - Thought it was helpful that the young people and support workers were all treated the same and were asked to just introduce themselves with their name and favourite sport (rather than their role at service); was useful to level the power dynamic - Enjoyed the flow and the gradual progression of activities - Expressed that the activities made it enjoyable for all skill levels; non-conventional sporting activities (e.g., tag) useful to build confidence and foster engagement for those with lower sport skill levels - Enjoyed the confidence metre to help participants relate to one another - Pedometers were well received   Community worker phone interview (primary support worker of 1 participant):   - Expressed that the icebreaker was useful to make people feel comfortable - Expressed that the session catered to all levels well- activities allowed were age appropriate and allowed for graded participation - Game with whole group at the end was a favourite part of the participants and support workers   Group interview (with 2 peer support workers and 2 community workers):   - Progression worked well to foster engagement - Enjoyed the use of the watches - Enjoyed the food/snacks, iced water, and electrolytes- felt well cared for - Thought it was useful that the facilitators participated rather than observing/note taking - Felt that it went fast; didn’t feel like exercise and were amazed by the high step count - Having the support workers and facilitators involved levelled the playing field and normalised it - Young people expressed that the facilitators were friendly and non-judgmental - Noticed increased self-esteem and interaction amongst young people   Email response from project administration worker:   - “I think the general feedback on the program has been very positive so far from both clients and staff.” - “A few people have commented that they thought it was going to be a bit boring at first (ie: just playing basketball) and they were really happy that it involved lots of games which makes it a lot more engaging that “just playing sport”.” - “I think the warm ups and games really help facilitate that social aspect and gives people of a wide range of abilities a better chance to participate. I also think the warm up games better facilitate that social aspect of sport, it’s more interactive and fun – whereas during the basketball games, it can get competitive and people more skilled at basketball dominate the game.” - “I thought having participants physically move into spaces to indicate opinion (ie: rating yourself against the coloured cones, putting yourself into the box you agreed with) was a really useful activity. I think prompts people to gain a different insight into their beliefs when they’re asked to physically embody what they think – quite different from the “tick the box” forms we usually put clients through.” - “The activities we did at the last session were a lot of fun, and I think they were at an appropriate level for clients (and staff, aha).” - “(The facilitators) have been wonderful to work with and the clients all seem to really like the team!” | - Keep structure but progress to group game sooner if ready - Continue to participate as facilitators, rather than take observational notes during sessions - Encourage helping behaviours amongst participants - Provide rationale for activity components, especially if not overtly related to the sport of the day - Provide more education around pedometers (and HR monitors when introduced) - Continue to create challenged and constraints for activities to encourage graded participation; be sure to divide skills players amongst teams and provide constraints if necessary (e.g., can only have the ball for 3 seconds; must stay in one zone, etc) - Allow more time for group game and for informal social time at end - Have informal social time at the end outside or in a dedicated room at the sport centre (depending on weather) - Engage participants in some standing stretching activities during warm-up to avoid standing - Keep “housekeeping” component brief - Label food with ingredients (especially wraps and sandwiches) |
|  | **What could be better:**   - Skill learning felt a bit   Slow and basic when in pairs; could progress to more challenging activities and small groups sooner   - Participants seemed comfortable moving into the large group activities sooner than expected, and appeared to enjoy this the most- could get to this phase sooner - More constraints might be needed for skilled players. At times, two individuals with basketball expertise dominated the game. - It felt a bit rushed at the end- be sure to allow time for more social interaction - The food at the end was served in a small public area- would be better in a space with more room | **What could be better:**  Email response from young person:   - “I would like it if we played longer games and did more drills to better our basketball skills” | **What could be better:**  Community worker phone interview (primary support worker of 2 participants):   - Food/informal social time phase felt rushed and cramped - More education on the importance of upping your step count in relation to health etc would be useful - Would be useful to label food (e.g., sandwich ingredients) for those with certain restrictions (*note- dietary restrictions were asked about in consent session and catered for)   Community worker phone interview (primary support worker of 1 participant):   - Would be useful to use more basketball specific exercises in the warm-up, or to explain rationale behind specific warm-up exercises if there aren’t basketball related     Group interview (with 2 peer support workers and 2 community workers):   - Felt rushed at the end; would enjoy more time playing game at end and less time in the introduction phase - Some of the skill development (e.g., shooting into hula hoops) felt too basic - Was hard to hear the facilitators at times; especially for those hearing voices   Email response from project administration worker:   - “During the basketball games, it can get competitive and people more skilled at basketball dominate the game” - “Given it’s a study, I think the amount of talking and explaining is probably unavoidable. But trying to minimise the amount of time standing still in a circle would be an improvement? Perhaps we can have some those conversation while doing a light warm up or stretching activity could be a little more engaging.” |  |
| **Session 2**   - Indoor basketball - 4/6 participants attended - 9 support workers attended (3 peer support workers) | **What went well:**   - Built on rapport well; able to follow up with young people on conversations from last week and form deeper connections - Participants appeared to be more comfortable and at ease from beginning - Helpful to have 30 minutes for cool down and social time/snacks; didn’t feel rushed - Helpful to book designated room for informal social time/snacks (too hot to be outside) - Progression and flow went well; seemed to move from phase to phase naturally. Was helpful to provide rationale before each phase - A facilitator was able to engage with young person who chose to sit out for most of session; participant appeared to enjoy this and engage well - Participation levels were high | **What went well:**   - No participants responded to feedback requests | **What went well:**  Brief group interview in service team meeting:   - Staff and clients are enjoying sessions; looking forward to them - Designated room for informal social time at end was useful for interaction - Staff and clients enjoyed having more time on the court for both small and large group basketball activities/games - Progression and graded participation helpful for engagement and rapport - Young people are enjoying the vouchers; some are saving them up to buy something bigger - Providing transport helps with accountability | - Have music playing and arrival activities set up upon arrival; facilitators to help set up watches and engage participants in activities (e.g., beach bats, ring toss) while waiting for others to arrive - Check in with participants throughout to ensure watch is working - Provide more sport specific examples of life skills; embed throughout sessions |
|  | **What could be better:**   - Energy levels felt lower than session 1; especially low energy in intro - Life skills component (motivation) felt a bit too academic; could be integrated into sessions better | **What could be better:**   - No participants responded to feedback requests | **What could be better:**   - One young person struggled to get watch (pedometer) to work - More sport options in voting would be helpful (water sports and hockey suggested) - Motivation levels would be low without transport support - Music during informal social time would be helpful |  |
| **Session 3**   - Indoor touch rugby - 5/7 participants attended (2 new participants) - 5 support workers attended (0 peer support workers) | **What went well:**   - Group responded well to 2 new participants; existing participants were welcoming and friendly which was helpful (in addition to the support workers and facilitators) - A new sport that was relatively novel to all helped to level the playing field in terms of skills, and also seemed to make it an easier entry for new participants - It was helpful to actively engage young people who chose to sit out during activities in alternate activities (e.g., ring toss) | **What went well:**  Phone interview with young person   - Enjoyed whole session, found it fun and engaging - Suggested we keep doing what we are doing and “keep on keepin on” - Felt supported when chose to sit out because of headache | **What went well:**  Feedback form from support worker:   - Most enjoyable components: Warm-up, game, cool down, and social/snack time - Components most useful for recovery: The cool down and socialise phases. “All of my clients have been fairly socially isolated before joining our service, the study is a good time for them to socialise with peers” - “I thought it was beneficial choosing rugby as the skill level across the group was more evenly distributed”   Feedback form from support worker:   - Most enjoyable components: “The Team building and the skills part of the training. The journaling and the setting goals before the session and then after. (For the clients) The Team building exercises and working with their case manager with skills. Also exchanging skills regarding the exercises and drills.” - Components most useful for recovery: “The Tracking of steps- weight loss and exercise; healthy food offered afterwards- Weight loss and encouraging healthy eating; journaling- Setting goals and taking time to reflect on them after the session.” - My client has commented on how friendly and welcoming the students are running the programme and how she feels non-judged and comfortable while participating. | In session modifications:   - Planned for the session to be outside but moved inside due to weather; adjusted planned activities to be executed on a court rather than a field - At least one facilitator engaging with young people sitting out - Support young people choosing to sit out, but also check in and offer alternative activities - Followed up with two young people who sat out due to headache or knee pain the next day to make sure they were alright   Next session:   - Make social time at end and during breaks a priority - Encourage/model use of alternative activities (and encourage support workers to do the same) - Begin embedding opportunities for young people to lead/instruct where appropriate |
|  | **What could be better:**   - Was challenging to balance introducing new participants to the program and making them feel comfortable while also building existing group cohesion - 3 participants sat out for a majority of the session (1 because of a sore knee from a previous injury, and 1 because of a headache after taking a ball to the head in the warm-up). Facilitators and support workers checked in with both, and both reported that they were fine. 1 chose to engage in alternative activities provided (ring toss), while the other chose to engage with their phone but reintegrated with the group at the end of the session for the cool down/social phases. One of the new participants sat out briefly to catch breath, but then returned to the activities - Challenge: alleviating barriers that arise ‘in session’ e.g., (bad experience, lack of interest in sport, perception of group etc.) while remaining aware that a one size fits all program is impossible. | **What could be better:**   - Nothing reported | **What could be better:**  Feedback form from support worker:   - A full length social group following sports would be helpful for the clients’ recovery goals   Feedback form from support worker:   - Warm-ups were the least enjoyable component - “I think you do a really great job. I can’t think of any other ways you could support them better I think you have thought of everything 😊” |  |
| **Session 4**   - Outdoor touch rugby - 2/7 participants attended - 5 support workers attended (2 peer support workers) | **What went well:**   - facilitators were reflexive- adapted well to the small group size, heat, and low energy of the group (e.g., lower intensity activities, letting them continue activities they were enjoying, etc) - interaction levels with participants were high and felt natural - high level of engagement despite low numbers | **What went well:**   - No participants responded to feedback requests | **What went well:**  Feedback form from peer support worker:   - Most enjoyable components: “The warm-up sessions – pertaining to the shape-indicated balls and the game with the taggers and runners – were my most favoured parts of the session. There was lots of laughter and joking throughout.”   Feedback form from support worker:   - Most enjoyable components: “The rugby ball catch the tails game. Great one of the client at the group succeed throughout the game and have a very accomplished look on his face. Throughout the session both clients who attended seemed to thoroughly enjoy all components of the group. The warm up and cool down at the end of the session always appears popular with the clients and both clients seemed comfortable and relaxed as rapport has been built over the previous 4 sessions.” - Components most useful for recovery: “All social aspects of the group I feel are the most beneficial for our client’s goals as it provides an environment in which our clients can socialise in what feels like a more organic and natural setting for socialisation. (Rather than a formalised social group)” - “I feel the strength of this program, asides from the well-planned sessions, are the interpersonal skills of everyone that runs the study. A client commented to me on the way home that she will be sad to see this group end. When I suggested starting up a similar mixed sports group she agreed that would be good, but “it would not be as fun without (the facilitators)”. Structured yet casual delivery of this program has been very engaging for our clients, enabling some of them to engage in activities they otherwise would not, in a ‘youth friendly’ manner.” | In session:   - Adapted session to the small group size, heat, and low energy of the group (e.g., lower intensity activities, letting them continue activities they were enjoying, allowing more time in the shade for social interaction, etc)   For next session:   - General thoughts for next session/future sessions or programs: Find ways to create opportunities to foster individual goals that differ markedly from the holistic goals of the group. E.g., participant with desire to develop coaching/leadership. Activities could be developed with the opportunity to support/instruct others within games |
|  | **What could be better:**   - Session was on the Tuesday of Easter week which may have contributed to the low numbers | **What could be better:**   - No participants responded to feedback requests | **What could be better:**  Feedback form from support worker:   - “For future consideration. I feel the group had less participants due to the Easter break. A large majority of our clients have dropped out of school and work. Our clients have a lot of spare time whilst their peers work or study. The Easter break has been a time when their friends and family also have some time off, so I feel they chose to socialise with their peers over attending the group.”   Feedback form from peer support worker:   - “I noticed some of the other people (including a young person) began engaging a tiny bit less towards the proper Touch Rugby game too. Although the activities were a medium intensity and it was stated that this was preferable, perhaps consider looking into exchanging the intensity so there’s a mix of low intensity and medium intensity games, to decrease people getting worn out before the proper game starts” |  |
| **Session 5**   - Indoor field hockey - 3/6 participants attended - 6 support workers attended (2 peer support workers | **What went well:**   - High levels of social engagement; participants seemed comfortable and were organically engaging in conversation with each other and the facilitators and/or support workers - Participants seemed to be happy to pair with anyone in group - Group cohesion and rapport levels appeared high | **What went well:**   - No participants responded to feedback requests | **What went well:**  Feedback form from support worker:   - Most enjoyable components: “The Team sports at the end of the session, warm up drills, the socialising, and the friendly atmosphere.” - Components most useful for recovery: “Reflective journal writing and the breathing exercises to use when feeling overwhelmed and unable to concentrate.” - “I think it’s great that we are able to exercise in a friendly and supportive environment.”   Feedback form from support worker:   - Most enjoyable components: “Main game component at the end of the session and debrief/socialise with clients. Positive comments were made in the car on the way home regarding all aspects of the session. Warm up games were well received and clients felt more relaxed than previous weeks.” - Components most useful for recovery: “Physical health goals were mentioned in the car on the way home. One client commented that she sleeps better on the days which she engages in Play On and it has made her consider joining further sports clubs in an effort to improve her sleep and overall physical health” | In session:   - Constraints made for more experienced players (ie must keep cones on head while playing   For next session/future programs:   - Find more opportunities to embed life skills into sport session - Provide more rationale for life skills as it relates to the sport to normalise skills and increase buy in - Find more opportunities to provide leadership opportunities to young people (e.g., taking on role of captain, instructor role in ice breakers, decision making in team building activities) |
|  | **What could be better:**   - Engaging stronger players in supportive roles (i.e. neutral zones) - Lack of congruence of the life skills within the session; create more opportunity to embed life skills within sporting activities. - Finding the rationale behind players sitting out. - Some players with lower levels of confidence appeared to be deterred by others playing with more intensity. - Although participants were more engaged socially, they were less engaged in the sport components | **What could be better:**   - No participants responded to feedback requests | **What could be better:**  Feedback form from support worker:   - “The amount of time spent on Ice breakers hence we are on week 5 of play on I felt it could be shorter as we all know each other.” - “I feel like giving the young people some responsibility and enabling them to take on some leadership roles would be useful.”   Feedback form from support worker:   - “Consider allowing some clients to take on leadership positions in sports. Eg. rotating team captains etc. This could help develop confidence and rapport within the group.” |  |
| **Session 6**   - Indoor field hockey - 1/6 participants attended - 6 support workers attended (2 peer support workers) | **What went well:**   - Adapted to low participant numbers. Young person did not seem to mind, and support workers participated as normal - Low intensity of activities led to more interaction and more team building components, opportunities to build team cohesion - More opportunities to encourage leadership roles (e.g. each person had to choose a strategy) | **What went well:**   - No participants responded to feedback requests | **What went well:**  Feedback form from support worker:   - Most enjoyable components: “The game where we sent different amounts of attackers/defenders depending on how many points we were after. It was good to see the client make some decisions and be involved in the strategy of the game. Unfortunately, none of my clients made it to the group this week. However, (the participant in attendance) appears more at ease in the group, more spontaneous in conversation with staff and play on facilitators and had built good rapport across the participants.” - Components most useful for recovery: “Socialisation in what feels like a more natural and organic setting. I feel that the Play On study provided a great structured yet informal way for our clients to socialise.” - “Elements and opportunities to delegate leadership to clients was a great way of involving and empowering them within the group. Building on interpersonal skills and confidence which they can take into their own life.”   Feedback form from peer support worker:   - Most enjoyable components: “The social interaction and the mini game where we had to choose the difficulty level and you didn’t know what the other team’s strategy was.” - Components most useful for recovery: “Team building and working together to play a sport. This promotes socialisation, having fun and exercise.” - “All of what we were working on together tied in really well and skills/teamwork we can all apply in life.” - “I though   Feedback form from peer support worker:   - Most enjoyable components: “Game of choosing which number of points to score/how many players to send in to attack/defend.” - Components most useful for recovery: “Physical activity, socialisation, reflecting on what they’ve gained from attending and how they can implement it in their life.” - “I thought it was an excellent program and very relevant/useful.” |  |
|  | **What could be better:**   - Session felt a bit slow in general- could have upped the intensity in warm up activities (lowest level of physical activity in warm up) - Difficult have final debrief as planned due to low participant numbers, but still useful to test components | **What could be better:**   - No participants responded to feedback requests | **What could be better:**  Feedback form from peer support worker:   - “Having a longer program? It went so quickly” - “Just having more of a consistent larger client base, which unfortunately we had no control over” |  |

Table S2

*Process Evaluation Using the MRC Guidance for Process Evaluation*

| **Function** | **Data Sources** | **Relevant Questions** | **Study Findings** | **Future Questions** |
| --- | --- | --- | --- | --- |
| **Description of intervention and its causal assumptions** | Development of a model through intervention mapping, including:   - narrative review - interviews with stakeholders (young people with FEP and their clinicians) - discussions with service providers - inclusion of relevant evidence based change mechanisms | What are the causal assumptions underpinning the intervention? | Study assumptions:   - Important components of FEP functional recovery include physical activity, social connectivity, and life skills development - Sport is a useful platform to foster physical activity, social connectivity, and life skills development - Therefore, a sport-based life skills program for young people with FEP should provide functional recovery benefits | - How can sport be maximised to best foster levels of physical activity, social connectivity, and life skills development in young people with FEP? - What program components yield long term benefits? - What types of sport are most conducive to functional recovery in FEP? |
| **Implementation** | - Participant feedback - Participant interviews - Facilitator observation and reflection notes | Implementation Process: How is delivery achieved (e.g., training, support, resources)? | - Facilitators had Masters level qualifications in sport and exercise psychology, and experience in playing and coaching sport, sport program delivery/development, and/or sport/exercise science or pedagogy - Facilitators received training re: FEP psychoeducation - The service provided critical support via design feedback, recruitment, transport, participation, and feedback during and after the intervention - The service reported that the overall burden was manageable and that the support provided was within their functional recovery role - The service reported that the time required for transport was manageable for the 6 weeks, but unsustainable long term. Suggestions from the service to mitigate this include: working with a more established service (a larger client base may result in less geographic spread of clients, and a more established relationship with the clients would enable conversations and planning for individual transport), and a longer program (to enable time for conversations/planning re individual transport) | - How can the support from the service be maximised, but the burden be minimised? - How can individual transport for the young people with FEP be supported? |
|  |  | What is delivered: | - The intervention was delivered as intended, with subtle modifications made throughout | - How can the intervention format be delivered in other formats (e.g., via other service providers, as a RCT) |
|  |  | - Fidelity: Was the intervention delivered as intended? |  |  |
|  |  | - Dose: What was the quantity of the intervention implemented? | - The intervention was offered once a week for 2 hours for 6 weeks - The service, facilitators, and participants all reported that this was not sufficient time for the secondary outcomes to be developed or measured | - What quantity of intervention will maximise functional recovery benefits? |
|  |  | - Adaptations: What adaptations were made in the implementation phase? | - Modifications included participation of all facilitators (rather than active observation/note taking), faster progression to group activities, and opportunity for participants to vote on sport utilised | - What adaptations need to be made to deliver the intervention in other formats (e.g., via other service providers, as a RCT, within other cultures or regions, with other sports) |
|  |  | - Reach: Does the intended audience come into contact with the intervention, and how? | - The reach extended only to clients of the service partner | - How can the reach be extended to other young people with FEP? |
| **Mechanisms of impact** | - Participant feedback - Participant interviews - Facilitator observation and reflection notes - Attendance and engagement records - Measurement engagement records and outcomes - Session plans/Modification records | How did the delivered intervention produce change? | - Alignment between program offerings and young people’s recovery goals fostered motivation - Environmental enablers fostered recruitment, attendance and engagement (i.e., structure, facilitators, staff participants)   - Evidence based change methods embedded in the structure promoted skill development and recovery methods - Logistical support (i.e., transport, food, reimbursement) enabled recruitment, attendance, and engagement - Attendance and engagement led to recovery benefits, skill development, and transfer (see results section for details) | - What components produced the most change? - How can change be sustained? - How can intervention components be more engaging and youth friendly? |
|  |  | How did the participants respond to and interact with the intervention? | - Attendance for young participants was 46.9% - When in attendance, participation levels were high for all participants - Feedback collected from young participants during and after the intervention was overwhelmingly positive - Feedback from staff participants suggested that participating in the program was positive and beneficial for the young people | - How can attendance and engagement be further supported? - How can we collect feedback from those who did not participate? - How can co-design be implemented more? |
|  |  | How did the facilitators impact the intervention? | - The participants reported that the welcoming and engaging nature of the facilitators was critical to engagement and normalisation - The participants and facilitators reported that inclusion of staff participants was critical for modelling and normalisation, and that they played an important role in creating a safe and supportive environment - The staff participants were critical for the recruitment and transport of young people | - What is the cost/benefit analysis of utilising multiple facilitators and staff participants? - How can young people be supported to facilitate their own transport? |
|  |  | What were the unexpected pathways and consequences? | - The value of the staff participants for modelling and normalisation was underestimated - The benefits for the therapeutic relationship between the young people and the service staff (e.g. rapport building and power differential balancing) were unexpected - The challenges of recruitment and retention were greater than expected - The challenges of working with a young service were greater than expected | - Will an established service be able to provide the necessary level of support? - What is the most beneficial facilitator to staff to young person ratio? |
| **Outcomes** | - Participant feedback - Participant interviews - Facilitator observation and reflection notes - Attendance and engagement records - Measurement engagement records and outcomes | Primary: Is a sport-based life skills program for young people with FEP feasible and acceptable? | - The results provide a compelling preliminary case for the feasibility and acceptability of a sport-based life skills for young people with FEP, and suggest that future work in this area is warranted - The results reveal the importance of partnering with a service for all phases of the intervention, and suggest that feasibility will be increased through collaboration with an established service | - Is a longer (e.g., 12 week) sport-based life skills program for young people with FEP feasible and acceptable? - Are there other sport based formats that would be more feasible/acceptable? |
|  |  | Secondary: Did the participants demonstrate changes in:   - Life skills development - Physical activity levels - Social engagement levels - Psychosis recovery? | - The results indicate a positive trend in the secondary outcomes, and call for further investigation (see the results section for more details) | - Does a sport-based life skills program for young people with FEP produce changes in:   - Life skills development   - Physical activity levels   - Social engagement levels   - Psychosis recovery? |
| **Context** | - Participant feedback - Participant interviews - Facilitator observation and reflection notes | How did context impact implementation and outcomes? | - The research study format (as opposed to a general functional recovery program provided by the service) may have impacted recruitment levels - The large catchment area of the service strengthened recruitment and transport challenges - The newness of the service limited recruitment and retention (e.g., because of low client numbers and unestablished therapeutic relationships with clients) - The short length of the intervention limited the development of secondary outcomes | - How does working with an established service impact recruitment, retention, and outcomes? - How does a longer program impact recruitment, retention, and outcomes? |

Table S3

*Process Evaluation Using the Bowen et al.’s (2009) Framework for Feasibility Studies*

| **Area of focus** | **Data Sources** | **Relevant questions** | **Study Findings** | **Future Questions** |
| --- | --- | --- | --- | --- |
| **Acceptability** | - Participant feedback - Participant interviews - Facilitator observation and reflection notes | To what extent was the program judged as suitable, satisfying, or attractive to participants? | - Feedback collected from young participants during and after the intervention was overwhelmingly positive - Feedback from staff participants suggested that participating in the program was positive and beneficial for the young people | - How can we maximise the program enjoyment? - How can the program be marketed to make it more attractive? - How can young people with FEP be more involved in the design process? |
| **Demand** | - Participant feedback - Participant interviews - Attendance and engagement records | What is the demand for such a program? | - Participants reported the need for a program that promotes physical activity, social connection, and life skills development in an engaging and normalised way - Participants reflected on the novelty of this program, and how it is unlike any other resources they have or have been offered | What regions, services, and/or phases of recovery are most in demand of such a program? |
| **Implementation** | - Participant feedback - Participant interviews - Facilitator observation and reflection notes - Attendance and engagement records - Modification records | To what extent can the program be successfully delivered? | - Successful delivery was enabled through:   - rigorous program design process (intervention mapping)   - support from the service   - qualified facilitators   - flexible design | - How can the support from the service be maximised, but the burden be minimised? - How can stakeholders be further involved in the implementation process? |
| **Practicality** | - Participant feedback - Participant interviews - Facilitator observation and reflection notes - Attendance and engagement records | To what extent can the program be carried out with intended participants using existing means, resources, and circumstances and without outside intervention? | - Outside intervention was utilised by way of bringing an external program to an existing service. | - How could existing services implement the program with existing resources? - What collaborations could enable limited outside resource use (e.g., university and service)? |
| **Adaptation** | - Participant feedback - Participant interviews - Facilitator observation and reflection notes - Session plans/Modification records | To what extent does the program perform when changes are made for a new format or with a different population? | - N/A | - What adaptations need to be made to deliver the intervention in other formats (e.g., via other service providers, as a RCT, within other cultures or regions, with other sports)? |
| **Integration** | - Participant feedback - Participant interviews - Facilitator observation and reflection notes - Session plans/Modification records | To what extent can the program be integrated within an existing system? | - The program was integrated into the functional recovery resources of an existing service | - How could a long term program be integrated sustainably into existing services? |
| **Expansion** | - Participant feedback - Participant interviews - Facilitator observation and reflection notes - Session plans/Modification records | To what extent can a previously tested program be expanded to provide a new program? | - N/A | - How can a full-scale version of the program be implemented? |
| **Limited efficacy** | - Participant feedback - Participant interviews - Facilitator observation and reflection notes - Attendance and engagement records - Measurement engagement records and outcomes | Does the program show promise of being successful with the intended population, even in a highly controlled setting? | - The results indicate that the program could be successful with the right service partner and facilitators, and design catered specifically to the population | - Would a full-scale version of the program yield functional recovery benefits? |
