# Supplementary material for: Enhancing functional recovery for young people recovering from first episode psychosis via sport-based life skills training: outcomes of a feasibility and pilot study
Source: Health Psychol Behav Med. 2022 Nov 21;10(1):1136–58. doi: 10.1080/21642850.2022.2147073 (PMC9683043; doi:10.1080/21642850.2022.2147073)
Supplement: Supplemental Material [file RHPB_A_2147073_SM7104.zip › HPBM-2022-0007_supp_figures.docx]

*Figure S1.* Pre and post results for the International Physical Activity Questionnaire (IPAQ) for 3 young participants. Display is in minutes of various types of physical activity in the past week (not an intervention week).

*Figure S2.* Pre and post results for the Basic Psychological Need Satisfaction and Frustration Scale (BPNSFS) for 3 young participants. Note: lower scores for needs frustration and higher scores for needs satisfaction are optimal.

*Figure S3.* Pre and post results for the Recovery Assessment Scale- Domains and Stages (RAS-DS) for 3 young participants. The maximum possible value is in parentheses.

*Figure S4.* Results for the Life Skills Scale for Sport (LSSS) for 3 young participants (delivered post intervention only).
